# Psychosocial interventions targeting mental health in pregnant adolescents and adolescent parents: a systematic review

**DOI:** 10.1186/s12978-020-00913-y

**Published:** 2020-05-14

**Authors:** Christina A. Laurenzi, Sarah Gordon, Nina Abrahams, Stefani du Toit, Melissa Bradshaw, Amanda Brand, G. J. Melendez-Torres, Mark Tomlinson, David A. Ross, Chiara Servili, Liliana Carvajal-Aguirre, Joanna Lai, Tarun Dua, Alexandra Fleischmann, Sarah Skeen

**Affiliations:** 1 Institute for Life Course Health Research, Department of Global Health, Stellenbosch University, Tygerberg, South Africa; 2 Peninsula Technology Assessment Group, University of Exeter, Exeter, UK; 3 School of Nursing and Midwifery, Queens University Belfast, Belfast, UK; 4 Department of Maternal, Newborn, Child and Adolescent Health and Ageing, World Health Organization, Geneva, Switzerland; 5 Department of Mental Health and Substance Use, World Health Organization, Geneva, Switzerland; 6 Data and Analytics Section, Division of Data, Analysis, Planning and Monitoring, UNICEF Headquarters, New York, USA; 7 Maternal, Newborn and Adolescent Health Unit, Health Section, UNICEF Headquarters, New York, USA

**Keywords:** Adolescent pregnancy, Adolescent parenthood, Mental health, Psychosocial interventions, Systematic review, Meta-analysis

## Abstract

**Background:**

Pregnancy and parenthood are known to be high-risk times for mental health. However, less is known about the mental health of pregnant adolescents or adolescent parents. Despite the substantial literature on the risks associated with adolescent pregnancy, there is limited evidence on best practices for preventing poor mental health in this vulnerable group. This systematic review therefore aimed to identify whether psychosocial interventions can effectively promote positive mental health and prevent mental health conditions in pregnant and parenting adolescents.

**Methods:**

We used the standardized systematic review methodology based on the process outlined in the World Health Organization’s *Handbook for Guidelines Development.* This review focused on randomized controlled trials of preventive psychosocial interventions to promote the mental health of pregnant and parenting adolescents, as compared to treatment as usual. We searched PubMed/Medline, PsycINFO, ERIC, EMBASE and ASSIA databases, as well as reference lists of relevant articles, grey literature, and consultation with experts in the field. GRADE was used to assess the quality of evidence.

**Results:**

We included 17 eligible studies (*n* = 3245 participants). Interventions had small to moderate, beneficial effects on positive mental health (SMD = 0.35, very low quality evidence), and moderate beneficial effects on school attendance (SMD = 0.64, high quality evidence). There was limited evidence for the effectiveness of psychosocial interventions on mental health disorders including depression and anxiety, substance use, risky sexual and reproductive health behaviors, adherence to antenatal and postnatal care, and parenting skills. There were no available data for outcomes on self-harm and suicide; aggressive, disruptive, and oppositional behaviors; or exposure to intimate partner violence. Only two studies included adolescent fathers. No studies were based in low- or middle-income countries.

**Conclusion:**

Despite the encouraging findings in terms of effects on positive mental health and school attendance outcomes, there is a critical evidence gap related to the effectiveness of psychosocial interventions for improving mental health, preventing disorders, self-harm, and other risk behaviors among pregnant and parenting adolescents. There is an urgent need to adapt and design new psychosocial interventions that can be pilot-tested and scaled with pregnant adolescents and adolescent parents and their extended networks, particularly in low-income settings.

## Plain English summary

While mental health during and after pregnancy has gained recognition as an important public health concern, adolescents who are pregnant or new mothers have been largely excluded from these conversations. Pregnant adolescents may experience stigma, higher rates of unplanned pregnancies, more physical health risks, and challenging social environments. Despite knowing the risks with adolescent pregnancy, there is limited understanding of how best to support this vulnerable group. This systematic review therefore aimed to identify if interventions using a psychological, behavioral, and/or social approach—psychosocial interventions—can effectively support the mental health of pregnant and parenting adolescents. To review the evidence, databases such as PubMed/Medline, PsycINFO, ERIC, EMBASE and ASSIA were searched. Seventeen eligible studies were included for the analysis. The results of this study showed that these types of interventions had a positive effect on positive mental health and school attendance outcomes. However, there was limited or no evidence for other outcomes such as parenting skills, substance use, self-harm and suicide or exposure to intimate partner violence. Only two studies included adolescent fathers, and no studies were based in low-income countries. The findings of this study show that there is a critical gap in knowledge about effective interventions for pregnant and parenting adolescents. There is an urgent need to adapt and/or design new interventions that can be tested and delivered to pregnant adolescents and adolescent mothers, fathers, and their extended networks, particularly in low-income settings, with a clear aim towards improving mental health outcomes.

## Background

The mental health of pregnant women and new mothers has long been a focus in the field of reproductive health [1–3]. However, less attention has been paid to pregnant adolescents and adolescent parents, despite evidence showing that adolescent girls and young women are at greater risk for developing mental health problems during pregnancy and after they give birth [4–7]. There is a growing imperative to focus on the needs of this vulnerable group. The global adolescent birth rate (ages 15–19) has fallen over the past two decades—from 56 births/1000 to 43.9/1000—but this decrease has been slower in low- and middle-income countries (LMICs) [8]. In an analysis of Demographic and Health Survey data from 30 LMICs, the percentage of all live births occurring to adolescents varied across countries from less than 10 to 33%, with a median of 18% for adolescents under 20 years of age [9]. To be able to support the health and wellbeing of pregnant adolescents and adolescent parents on a global level, it is critical to have a multifaceted understanding of their needs and challenges.

Pregnant adolescents and adolescent parents are vulnerable to poor mental health outcomes for three main reasons. Adolescence is a transitional stage characterized by psychological, biological, and social changes. Pregnancy and parenting during this critical period interferes with normative developmental processes [10], and the dual biological transitions of adolescence and pregnancy may increase individuals’ psychological and physical vulnerability [11]. The potential effect of these neurobiological changes can be observed in adolescent mothers experiencing higher rates of depression [6, 7], anxiety, and stress than older mothers. Pregnant adolescents and adolescent mothers also undergo social changes that may link to poor mental health outcomes. Adolescent mothers commonly overestimate the amount of support they will receive after childbirth, leading to increased stress and depression postpartum [4, 12]. Pregnancy, as a visible marker of early sexual activity, may lead to stigma, discrimination, and blame that can negatively affect adolescent mothers’ psychosocial wellbeing and isolate them at a time when they require more social support [13–15].

Second, alongside the risks related to the transition to adulthood and parenthood, adolescent girls face physical changes and potential health complications associated with early pregnancy and childbirth. These complications may elevate stress levels in new mothers, or make them vulnerable to mood disorders. Risks specific to adolescent childbearing include a higher likelihood of obstetric fistula [16] and of delivering a preterm or low birth weight infant [6, 12, 17]. These challenges may trigger or compound existing vulnerability to mental ill-health; conversely, psychosocial distress may make it difficult for pregnant adolescents and young mothers to care for themselves and their infants, leading to poorer health outcomes for mother and child [18].

Finally, adolescent pregnancy often occurs within environments of risk, which expose young women to multiple drivers of psychosocial distress and barriers to accessing care and support [19]. As many as two-thirds of adolescent pregnancies may be unintended [12, 20, 21], introducing new responsibilities and demands for adolescents who may not be ready to become parents, or may undergo additional stressors when experiencing an unintended pregnancy [22]. Furthermore, many pregnant adolescents are involved in age-disparate relationships, which may or may not continue following the pregnancy [23]. Adolescents who become pregnant outside the context of marriage may also be heavily stigmatized and prevented from accessing a broader support network [24]. Research suggests a bidirectional relationship between risk factors and pregnancy: while poverty and vulnerability increase the risk for early pregnancy and parenthood, adolescent parenthood can also lead to increased vulnerability [4, 12, 25, 26]. The same risk factors that may drive adolescent pregnancy—such as school dropout, substance use, partner or household violence [27], early marriage [28], and economic deprivation—can be exacerbated following pregnancy and childbirth. Importantly, although there are specific social, biological, and environmental risk factors for pregnant and postpartum adolescent girls and young women, there are additional mental health risks for adolescent fathers, including increased stress, economic deprivation, and adoption of risk behaviors [29], about which little is documented.

Identifying the role of mental health within these contexts is critical to disentangling the risks that young parents face: poor mental health can complicate an adolescent’s ability to be resilient, re-enroll in school, plan future pregnancies, and earn an income. Adolescents who do not finish school may struggle to obtain employment and their families may be more susceptible to economic instability; they are also at risk for rapid repeat pregnancy [6, 12, 30, 31]. These factors may also have intergenerational effects: children of adolescent parents have been found to struggle with social and emotional behaviors and with academic performance and are more likely to become adolescent parents themselves [7, 25, 32, 33].

Given these risks, it is critical to identify how to prevent mental health challenges in this population. Despite the substantial literature of the risks associated with adolescent pregnancy and parenthood, there is limited evidence on the effectiveness of psychosocial interventions among pregnant adolescents and adolescent parents to guide best practice to support this vulnerable group [7]. Promoting skills for healthy functioning, and preventing the onset of mental health conditions, is a pressing need, especially given the fact that adolescents are more likely to face constraints in accessing appropriate medical and psychosocial care [4, 34]. While evidence-based interventions exist, there is a clear need to identify if psychosocial interventions can effectively support the mental wellbeing of pregnant adolescents and adolescent mothers and fathers—promoting positive mental health and preventing mental health disorders, risk behaviors, self-harm and suicide.

## Methods

This review was part of a larger set of systematic reviews informing the new WHO Guidelines on Mental Health Promotive and Preventive Interventions for Adolescents [35]. These guidelines aim to provide global, evidence-informed recommendations on psychosocial interventions for adolescents, delivered across a range of platforms, to promote positive mental health and prevent the development of mental disorders and risk behaviors. We used a standardized systematic review methodology based on the process outlined in the *WHO Handbook for Guidelines Development, Second Edition* [36]. The review protocol was registered on PROSPERO (CRD42019123723).

### Inclusion and exclusion criteria

This review included primary studies published in peer-reviewed journals between January 2000 and February 2019. We included evaluations of psychosocial interventions, which were defined as interventions adopting a psychological, behavioral, and/or social approach [37, 38], to improve psychosocial well-being and/or reduce the risk of poor mental health outcomes. To be included, these psychosocial interventions had to include as primary or secondary outcomes 1) the promotion of positive mental health (which includes measures related to mental wellbeing and mental functioning), 2) the prevention of mental disorders (depression and/or anxiety), and/or 3) the prevention of self-harm and suicide.

Participants were pregnant adolescents or adolescent parents (male and/or female), between 10 and 19 years of age, based on the WHO definition of adolescence [39]. Where studies included a broader age range, we included studies with a mean age, or with 50% or more of the sample, falling within this age range. Eligible programs could target adolescents individually or in groups or could target their caregivers and families but track adolescent outcomes.

We included randomized controlled trials (RCTs), crossover trials, cluster randomized controlled trials and factorial trials. These studies compared interventions to treatment as usual, referring to adolescents receiving the usual or routine treatment or care available to adolescents or no intervention. Studies comparing two active psychosocial interventions were removed from this analysis. Treatment intervention studies on adolescents with a diagnosed disorder were not included. No language or geographic restrictions were imposed on the sample.

### Search methods for identification of studies

We used a predetermined set of search terms to conduct systematic searches on PubMed/Medline, PsycINFO, ERIC, EMBASE and ASSIA (see Supplementary File 1 for the full search strategy). Studies from January 2000 to February 2019 were included. We also searched reference lists of relevant articles and grey literature and consulted experts in the field. All search results were exported to EndNote, where duplicates were removed, and then to EPPI-Reviewer, a web-based tool for systematic review management [40]. We applied a machine learning RCT classifier using EPPI-Reviewer, which assigned to each record the probability that it reported an RCT. Abstracts that were categorized as having at least a 20% likelihood of being an RCT were reviewed by two reviewers working in pairs against inclusion criteria. Those with less than 20% likelihood of inclusion were re-reviewed by one reviewer only, with the machine learning classifier acting as the second “reviewer.” This method has been adopted for improving systematic review accuracy and combining human and machine productivity [41]. To ensure no studies were missed, key search terms were also used to search through abstracts. All reviewers convened to discuss discrepancies between reviewers. Following this, full-text versions of studies that were deemed to be potentially eligible for the review were obtained and two reviewers independently evaluated each paper. In cases of doubt, the full text article was subjected to adjudication by a third researcher on the team.

### Data extraction

Study characteristics (setting, population, research design, intervention details, screening tools) were entered into an MS Excel database by two reviewers. The first author independently reviewed each extraction for quality control purposes. Outcomes were extracted by one reviewer and all entries were independently checked by another reviewer. Outcome-related data include type of control group, outcome category, instrument, timepoint, sample size of intervention and control groups, intervention effects (e.g., means, standard deviations), a calculated standard mean difference (SMD) and 95% confidence intervals [42]. We contacted study authors via email for any missing data, and incorporated responses that were received within our timeframe. We combined data from studies with multiple publications into one study record. When studies had more than two arms, active interventions were compared to treatment as usual, but not to one another.

#### Types of outcome measures

We included studies where the primary focus was 1) promoting positive mental health, 2) preventing symptoms as well as incidence of depression and/or anxiety, and/or 3) preventing self-harm and suicide. Positive mental health included mental wellbeing (with measures such as quality of life and self-esteem) and mental functioning (with measures such as resilience, problem-solving, and emotional regulation). Each eligible study had to include at least one of these three outcomes. Other outcomes extracted included substance use (including alcohol, smoking, and drugs); aggressive, disruptive, and oppositional behaviors; risky sexual and reproductive health behaviors; school attendance; adherence to antenatal and postnatal care; parenting skills (knowledge, attitudes, and behaviors); and exposure to intimate partner violence.

#### Risk of bias

We used the Cochrane Risk of Bias tool to assess risk of bias and study quality [43]. This tool included random sequence generation and allocation concealment (selection bias); blinding of participants and personnel (performance bias); blinding of outcome assessment (detection bias); incomplete outcome data (attrition bias); selective reporting (reporting bias); and other sources of bias. All risk of bias assessments were made independently by two reviewers. After agreement on any discrepancies, the senior independent reviewer recorded all changes and corrections. The quality of evidence was judged using the Grading of Recommendations, Assessment, Development, and Evaluation (GRADE) methodology [44]. Quality control meetings were held approximately weekly to address issues as they arose. In preparing for GRADE, we also investigated the potential of publication bias in the distribution of our outcomes of interest using funnel plots plotting standard errors against effect sizes, where appropriate (i.e. when there were at least 10 studies contributing data to a given outcome).

### Data analysis

Outcome data across all time points were combined for the meta-analyses. Effect estimates from included studies were categorized according to the outcome domain (e.g., positive mental health, mental disorders, substance use or school attendance) they represented and length of follow-up. Individual effect estimates were transformed into standardized mean differences (SMD, i.e., Cohen’s d with 95% Confidence Intervals [CI]) [45]. Effect estimates of 0–0.2 were considered small, 0.2–0.5 considered small-to-moderate, 0.5–0.8 considered moderate-to-large, and 0.8 and above, large. Where binary outcomes were reported, odds ratios were converted to Cohen’s d using the logit transformation.

We used robust variance estimation with random effects to account for multiple dependent effect estimates per study during the meta-analysis (for example, where one study contributed several effect estimates to one outcome domain). An assumption of an intercorrelation of 0.8 within studies was made. Heterogeneity was described in terms of τ^2^ adjusted for clustering and I^2^.

Meta-regression included categorical predictors to describe intervention and population characteristics that may account for heterogeneity in effectiveness, shared below. Meta-regressions were described using the regression coefficient, residual I^2^, and residual τ^2^. We conducted sensitivity analyses to assess any differences between intervention effect by gender, and by whether or not a mental health-based screening tool had been used to select participants into the intervention. The meta-analyses were conducted using Stata v.16.

## Results

**Fig. 1:**
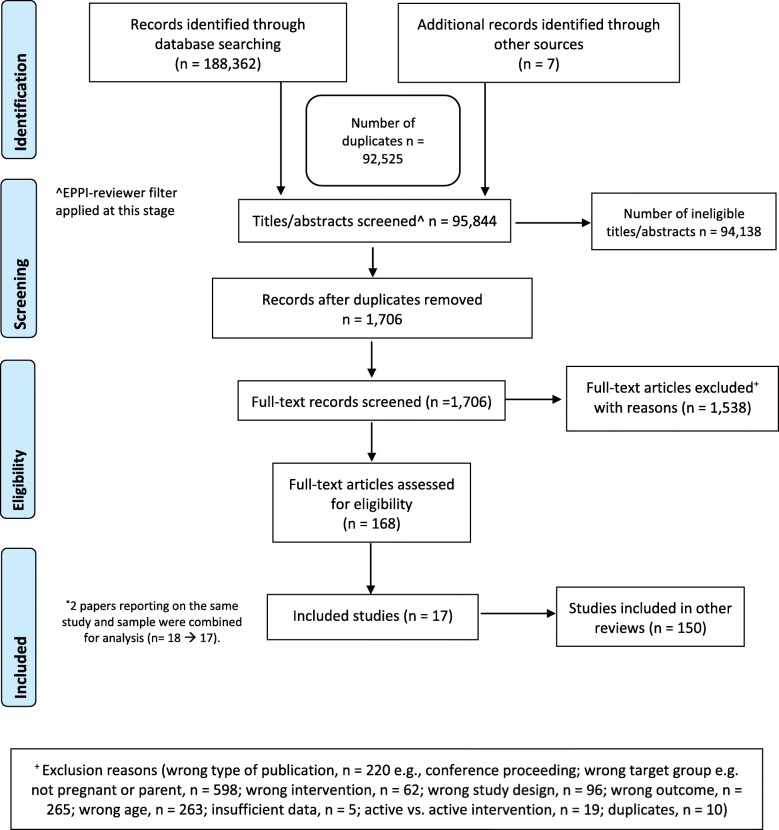
Flow chart of included studies

Overall, 95,844 de-duplicated citations were identified through the database search. The high number of citations was due to the fact that we ran multiple searches concurrently. After screening of titles and abstracts, the full texts of 1699 articles were screened (Fig. 1). We excluded 1538 full-text articles based on reasons such as incorrect target group/age, intervention type, or incorrect study design. We allocated an additional 150 studies to other research questions included in this search strategy that belonged to other reviews under the *WHO Guidelines on Mental Health Promotive and Preventive Interventions for Adolescents*. This resulted in 17 included studies (*n* = 3245 participants) comparing psychosocial interventions for pregnant adolescents and adolescent parents with treatment as usual.

### Study characteristics

Studies were conducted in three high-income countries in the region of the Americas: the United States (*n* = 14 studies [33, 46–59];, Canada (*n* = 2 [60, 61];, and Chile (*n* = 1 [62]). A short description of all studies is given in Table 1. Individually randomized controlled trial designs were used to evaluate the majority of intervention studies (*n* = 15); the remaining two used a cluster RCT and a factorial trial design.

**Table 1:** Included studies

Author and year	Country	Program Intent	Study Design	Total sample (N)	Age (mean, SD)	Females (%)	Males (%)	Study population description	Outcomes reported						
									Positive mental health	Mental health disorders	Substance use	Risky sexual and reproductive health behaviors	School attendance	Adherence to ante-natal and post-natal care	Parenting skills
Aracena et al. (2009) [62]	Chile	Prevent depression; promote family functioning	RCT	90	17.21, 1.38	100%	n/a	Young women pregnant for the first time, living in an extremely poor neighborhood of Santiago		X					X
Barlow et al. (2013); Barlow et al. (2015) [51] [52]	U.S.A.	Prevent depression, stress, and substance use; promote parenting knowledge	RCT	322	18.12, 1.47	100%	n/a	Pregnant (= < 32 weeks gestation) American Indian adolescents residing in one of four participating reservation communities		X	X				X
Barnet et al. (2002) [33]	U.S.A.	Prevent depression, anxiety, and stress	RCT	232	16.0, n/s	100%	n/a	Pregnant or recently delivered (< 6 m) attending an alternative school specifically for childbearing adolescents; predominantly low-income and African-American		X					X
Barnet et al. (2007) [49]	U.S.A.	Prevent depression and repeat pregnancy; promote parenting behaviors, contraceptive use, and school enrolment	RCT	84	16.9, 1.4	100%	n/a	Pregnant adolescents, predominantly low-income African American; fathers of infants were invited with mother’s consent		X		X	X	X	X
Felder et al. (2017) [55]	U.S.A.	Prevent depression	cRCT	1233	18.64, n/s	100%	n/a	Pregnant adolescents < 24 weeks gestation		X					
Florsheim et al. (2012) [47]	U.S.A.	Promote parenting behaviors, relationship quality, and positive interactions	RCT	210 (105 couples)	16.5 (mothers); 18.5 (fathers)	50%	50%	Pregnant adolescents < 26 weeks gestation and their co-parenting partners (biological fathers of children)	X						X
Ginsburg et al. (2012) [46]	U.S.A.	Prevent depression; promote social support	RCT	47	18.15, n/s	100%	n/a	Pregnant adolescent Apache American Indian women < 28 weeks gestation; CES-D score of 16 or higher at baseline	X	X					
Harris & Franklin (2003) [57]	U.S.A.	Promote problem solving and school attendance	RCT	86	17.93, n/s	100%	n/a	Adolescent women who were pregnant (<8th month) or currently parenting one or more children of whom they had custody	X				X		
Koniak-Griffin et al. (2002) [50]	U.S.A.	Promote self-efficacy	RCT	102	16.78, 1.12	100%	n/a	Underserved first-time pregnant mothers	X						
Kumar et al. (2016) [60]	Canada	Prevent depression	RCT	30	17.4, 1.2	100%	n/a	Mother-child dyads		X					
Logsdon et al. (2005) [53]	U.S.A.	Prevent depression; promote social support and self-esteem	Factorial RCT	109	16.0, 1.3	100%	n/a	Pregnant and parenting adolescents	X	X					
Mazza (2002) [48]	U.S.A.	Promote relationships and birth control	RCT	60	16–18	n/a	100%	First-time fathers, African-American, between 16 and 18	X					X	
McDonell et al. (2007) [58]	U.S.A.	Prevent substance use and risk for STD; promote self-efficacy, social support, and problem solving	RCT	197	17.5, n/s	n/s	n/s	Low-income pregnant and parenting teens	X		X	X			
Phipps et al. (2013) [59]	U.S.A.	Prevent depression	RCT	106	16.0, n/s	100%	n/a	Pregnant and parenting adolescents who conceived first pregnancy at 17 or younger		X					
Samankasikorn et al. (2016) [56]	U.S.A.	Prevent depression and stress; promote self-esteem and social support	RCT	150	17.31, n/s	100%	n/a	Pregnant teens	X	X					X
Stirtzinger et al. (2002) [61]	Canada	Prevent depression; promote parental efficacy and perceived control	RCT	20	17.0, n/s	100%	n/a	Adolescents who were pregnant or parenting young children; only including those with a score of 16 or above on Beck’s Depression Inventory (akin to clinically depressed but no other diagnostic tool used)		X					X
Walkup et al. (2009) [54]	U.S.A.	Prevent depression, stress, and substance use; promote parenting knowledge, maternal involvement and social support	RCT	167	12–22	100%	n/a	Expectant American Indian adolescents at 28 weeks gestation or less	X	X	X				X

*RCT* Randomized controlled trial; *cRCT* Cluster randomized controlled trial; *n/a* Not applicable; *n/s* Not specified

Sample size ranged from 20 to 1233 participants (mean = 190, median = 106). Almost all studies (*n* = 15) reported on participants’ mean age; the remaining two studies reported age range. All but one study recruited adolescents aged 15 or older; the other study included participants from ages 12–22. Fifteen studies included only female participants; one study included both males and females [47], and one included only males [48]. Two studies included mental health screening tools to select participants for recruitment [46, 61].

The number of studies that reported each outcome is shown in Table 2. Most studies reported on mental disorders (*n* = 12), and around half reported on positive mental health (*n* = 9) and/or parenting skills (*n* = 8). None of the studies measured self-harm and suicide; aggressive, disruptive and oppositional behaviors; or exposure to intimate partner violence (IPV).

**Table 2:** Overall effect sizes per outcome

	All time points				
	Effect size	***p***-value	95% Confidence Intervals		Heterogeneity (I^**2**^)
Positive mental health (*n* = 9)	0.35	0.01**	0.10	0.61	75%***
Mental disorders (depression and anxiety; *n* = 12)	−0.11	0.21	−0.30	0.08	59%**
Self-harm and suicide (*n* = 0)					
Aggressive, disruptive and oppositional disorders (*n* = 0)					
Substance use (*n* = 3)	*−0.27*	*0.26*	*−1.10*	*0.56*	61%*
Risky sexual and reproductive health behaviors (*n* = 2)	*−0.17*	*0.56*	*−2.68*	*2.35*	60%
School attendance (*n* = 2)	*0.64*	0.01**	*0.55*	*0.72*	0%
Adherence to antenatal and postnatal care (*n* = 2)	*0.31*	*0.53*	*−4.04*	*4.66*	35%
Parenting skills (*n* = 8)	*0.07*	*0.47*	*−0.16*	*0.30*	71%**
Exposure to IPV (*n* = 0)					

*p < 0.05; **p < 0.01; ***p < 0.001. Models in italics are indicative only, given the statistical estimation procedures used. For positive mental health, school attendance, adherence to antenatal and postnatal care, and parenting skills, a positive effect size denotes a beneficial effect. For all other outcomes, a negative effect size denotes a beneficial effect

### Intervention implementation

Participants’ homes were the most common location of intervention (*n* = 7). Three studies were conducted in health centers, two in school settings, two in community settings, and three in a combination of these settings. Programs were most commonly implemented by lay health workers (*n* = 7), though some were implemented by mental health professionals (*n* = 3), other health professionals (*n* = 2), or a mixed team of professionals (*n* = 2). One study was delivered through video- and pamphlet-based content, and the two remaining studies did not specify the type of implementer.

The face-to-face interventions were delivered individually (*n* = 10), in groups (*n* = 3) or in some combination of both (*n* = 3). Of the 15 studies reporting intervention contact time, time ranged from 4 to 43 h, with a mean of 18 h. Five studies followed up participants over a longer duration, continuing from 6 months to 2 years postpartum; 12 studies had shorter follow-ups. Less than a third of studies (*n* = 5) explicitly reported that adolescents were involved in developing the intervention. More than half of the studies (*n* = 10) were individualized and tailored to participants’ individual needs and preferences. These included studies that were conducted with expectant and recently-delivered adolescent mothers at home, in one-on-one settings, and sought to address individual challenges faced, as well as other community-based programs that met with participants individually to identify avenues for enhanced support.

### Risk of bias

The method for generating a randomization sequence was reported acceptably in less than half of studies (*n* = 7, 41.1%), with the balance judged at unclear risk of bias. Only two studies reported explicitly on allocation concealment; the other studies were assessed as having an unclear risk of bias (*n* = 15, 88.2%). The domains with the most high-risk judgments were incomplete outcome data (*n* = 5, 29.4%) blinding of participants and personnel (*n* = 4, 23.5%), and blinding of outcome assessment (*n* = 4, 23.5%). There was a low risk of bias for selective reporting in the majority of studies (*n* = 15, 88.2%). Other biases posing high risk were identified in three instances: these related to loss of a cluster in one cluster RCT [55], potential volunteer bias [56] and baseline non-equivalence [51, 52, 56]. Risk of bias assessments for each article are available in Supplemental File 2.

### Meta-analysis results

Collectively, psychosocial interventions for pregnant adolescents and adolescent parents showed important, small to moderately sized beneficial effects on positive mental health (SMD: 0.35, 95% CI: 0.10, 0.61; nine RCTs, *n* = 1539) compared to treatment as usual and averaged over all time points post-intervention (Table 2). We identified substantial statistical heterogeneity between studies with an *I*^*2*^ of 75% for this outcome.

School attendance outcomes demonstrated moderate-to-large beneficial effects of psychosocial interventions (SMD: 0.64, 95% CI: 0.55,0.72; two RCTs, *n* = 170). However, results for school attendance are indicative only, given the requisite sample sizes for robust variance estimation and the small number of trials (*n* = 2). No statistical heterogeneity was evidenced. There were no available data on self-harm and suicide; aggressive, disruptive, and oppositional behaviors; or exposure to IPV.

Separate meta-analyses were conducted to test for differential intervention effects. These sensitivity analyses considered gender and if participants were screened into the study using mental health screening (i.e., to screen for postnatal depression). Results for primary outcomes were robust to these analyses (see Tables 3 and 4).

**Table 3:** Overall effect sizes per outcome, gender sensitivity analysis (studies with male participants excluded, *n* = 15 remaining)

	All time points			
	Effect size	p-value	95% Confidence Intervals	
Positive mental health	0.33	0.05*	0.00	0.66
Mental disorders (depression and anxiety)	−0.11	0.21	−0.30	0.08
Self-harm and suicide				
Aggressive, disruptive and oppositional disorders				
Substance use	*−0.27*	*0.26*	*−1.10*	*0.56*
Risky sexual and reproductive health behaviors	*−0.17*	*0.56*	*−2.68*	*2.35*
School attendance	*0.64*	0.01**	*0.55*	*0.72*
Adherence to antenatal and postnatal care				
Parenting skills	0.06	0.65	−0.24	0.25
Exposure to IPV				

*p < 0.05; **p < 0.01; ***p < 0.001. Models in italics are indicative only, given the statistical estimation procedures used. For positive mental health, school attendance, adherence to antenatal and postnatal care, and parenting skills, a positive effect size denotes a beneficial effect. For all other outcomes, a negative effect size denotes a beneficial effect

**Table 4:** Overall effect sizes per outcome, screening sensitivity analysis (screen-in studies excluded, *n* = 15 remaining)

	All time points			
	Effect size	p-value	95% Confidence Intervals	
Positive mental health	0.41	0.01**	0.15	0.66
Mental disorders (depression and anxiety)	−0.10	0.25	−0.31	0.10
Self-harm and suicide				
Aggressive, disruptive and oppositional disorders				
Substance use	*−0.27*	*0.26*	*−1.10*	*0.56*
Risky sexual and reproductive health behaviors	*−0.17*	*0.56*	*−2.68*	*2.35*
School attendance	*0.64*	0.01**	*0.55*	*0.72*
Adherence to antenatal and postnatal care	*0.31*	*0.53*	*−4.04*	*4.66*
Parenting skills	0.07	0.47	−0.179	0.33
Exposure to IPV				

*p < 0.05; **p < 0.01; ***p < 0.001. Models in italics are indicative only, given the statistical estimation procedures used. For positive mental health, school attendance, adherence to antenatal and postnatal care, and parenting skills, a positive effect size denotes a beneficial effect. For all other outcomes, a negative effect size denotes a beneficial effect

We also investigated studies that were identified as having high risk of bias in more than 3 of 7 domains (*n* = 2 studies [51, 52, 61];). Imprecision in the effect estimates from these two studies suggested that our pooled estimates were robust to their inclusion.

All studies were assessed using GRADE. Confidence in the quality of evidence varied, but was low overall when all outcomes were considered together. There was high quality evidence for school attendance outcomes; moderate quality evidence for mental disorders and parenting skills outcomes; low quality evidence for adherence to antenatal and postnatal care outcomes; and very low quality evidence for positive mental health, substance use, and risky sexual and reproductive health behavioral outcomes. The three criteria used for assessing overall risk of bias during GRADE were whether randomization was adequately described, whether detection bias was minimized through blinding, and whether the proportion of outcome data that were incomplete was low and balanced across trial arms. To gauge imprecision, we used a clinically relevant effect size of 0.2. Funnel plots were constructed to assess publication bias; publication bias was only strongly suspected for positive mental health and substance use outcomes.

## Discussion

In this review, we synthesized and meta-analyzed randomized controlled trials of psychosocial interventions to prevent and promote mental health for pregnant adolescents and adolescent parents. Our findings showed overall significant improvements in positive mental health and school attendance outcomes for intervention beneficiaries compared to controls. Although we undertook sensitivity analyses to gauge differential intervention effects by gender and mental health screening, these analyses did not suggest any changes in the pattern of results. Similarly, excluding the two studies with the highest identified risk of bias did not alter our pattern of results. We did not find evidence of the potential impact of risk of bias on the results. Positive mental health, with a focus on building healthy relationships and social support networks, may be a particularly important focus area for this developmental stage and for these adolescent mothers, especially in the context of other life challenges.

We identified substantial heterogeneity in positive mental health outcomes, indicating a diversity of studies contributing to this outcome. This heterogeneity might be attributable to differences in modes of intervention delivery or content of the psychosocial interventions delivered; it might also be explained by differences in types of positive mental health outcomes measured across the nine studies that included this outcome. Studies employing similar types of positive mental health measures might provide more homogeneous results.

No significant heterogeneity was observed for school attendance outcomes. Although interventions resulted in significant positive school attendance outcomes compared to controls, there were only two studies (of *n* = 17) that measured this outcome [49, 57]. More evidence is needed, therefore, on outcomes related to school re-enrollment, attendance, and completion to gauge the impact of psychosocial interventions on this vitally important domain [26, 63].

There was limited evidence for the effectiveness of preventive psychosocial interventions among pregnant adolescents and adolescent parents on symptoms and diagnoses of depression and anxiety, substance use, risky sexual and reproductive health behaviors, adherence to antenatal and postnatal care, and parenting skills. This may be in part because few studies reported on most of these outcomes, but our meta-analysis did not show significant benefits. Surprisingly, only two studies reported on risky sexual and reproductive health (SRH) behaviors, despite the clear link between sexual risk exposure and pregnancy/childbearing. It is plausible that our review eligibility criteria (including a mental health outcome) may have excluded other relevant studies on interventions to reduce pregnancy risk by targeting adolescent SRH. Psychosocial interventions for adolescents who are pregnant or parenting should specifically target SRH, as they could be beneficial in bolstering adolescents’ negotiating skills, improving confidence, and reducing overall rates of repeat pregnancy and sexually transmitted infections [25, 26].

Furthermore, there were no available data on outcomes related to aggressive, disruptive, and oppositional behaviors or exposure to IPV. Each of these outcomes represents a critical evidence gap within interventions for pregnant and parenting adolescents. There is also a need to explore the effect of violence and aggression. Adolescent girls experiencing IPV have been found to be at a heightened risk for becoming pregnant [64, 65]. A recent study found that experiences of bilateral violence (where pregnant adolescents both experience and perpetrate violence) had the largest effect on adverse mental health outcomes [66]. There were also no studies tracking self-harm and suicide. Pregnant adolescents and adolescent parents may be at a greater risk for suicidal ideation and self-harm [67], yet no interventions in our sample actively measured these outcomes. Interventions and evaluations incorporating these core areas are urgently needed in order to build evidence to better support young mothers, their partners, and their children [15].

Although the focus of this review was mental health, we noted that many included studies also contained specific provisions for improving parenting-related skills, to equip adolescent parents to be better caregivers. While we recognize the importance of this approach, it is also critical to focus on the psychosocial needs of these adolescents to more explicitly target improvements in their mental health outcomes [2, 4, 5]. We propose adaptations to existing interventions and the design of new interventions that employ more mental health-specific intervention content in order to meet the specific needs of adolescent mothers and fathers.

We did not restrict our search to female adolescents, but we uncovered only two studies that included male participants (adolescent fathers). Of these two studies, only one was for fathers only, published in 2002 [48], and the other was focused on co-parenting skills for partners expecting a child [47]. Apart from a parenting focus, there is a need for building skills and identifying modes of support for adolescent fathers, many of whom are marginalized and vulnerable in different ways to adolescent mothers. There is also a need for more programming that includes adolescent couples and co-parents, to improve mental health as well as communication and problem-solving skills.

Finally, the most notable omission in the studies that were identified by our review was that no randomized studies of psychosocial interventions were conducted in LMICs. This is pertinent considering that adolescents in LMICs are much likelier to be vulnerable to early, unintended pregnancy [30]. Despite geographic limitations, our current analysis of pregnant adolescents and adolescent parents has relevance for broader contexts: many studies used lay health workers to deliver interventions, and the majority of intervention beneficiaries were characterized as vulnerable, often mobile, and living in socioeconomically deprived circumstances [46, 49, 62]. Nevertheless, critical populations and global regions were missing from the evidence available to this review, and as such, only limited conclusions about the relevance and applicability of these psychosocial interventions in LMIC settings can be drawn.

A growing adolescent population, aided by large reductions in neonatal and child mortality, means that more adolescents than ever will be transitioning to adulthood in the next 30 years, especially in low-income settings [68]. In societies facing challenges with education, employment, health care access, and conflict, adolescent girls and young women face a higher probability of unintended pregnancies [21]. For this group, psychosocial interventions may be valuable for promoting positive mental health and preventing mental health problems and risk behaviors. Positive mental health in particular—emphasizing how to identify social support and nurture interpersonal relationships—may be an especially important area of focus for fostering mental health in these populations. They also have central roles to play in interrupting intergenerational cycles of risk and continued adversity [25, 27]. The lack of evidence from LMICs also prevents more nuanced understanding of co-existing health and social vulnerabilities in this population and how to appropriately address them. For example, data on high HIV incidence amongst adolescent girls and young women living in sub-Saharan Africa are readily available, as are data on adolescent pregnancy in the region; yet these data rarely consider both concurrently to indicate the scale of this phenomenon [69]. Early marriage is also nearly invisible from the literature on adolescent pregnancy, although a significant proportion of adolescent mothers are married [28, 30]. There is an urgent need to adapt and/or design new psychosocial interventions that can be pilot tested and scaled-up with pregnant adolescents and adolescent mothers, fathers, and their extended social networks in LMIC settings, with an explicit aim of improving mental health outcomes. In many LMICs where additional stressors may exist, new data from implementation efforts could signal where more programming is needed and how to best tailor it to diverse circumstances.

## Conclusion

We found that, in high-income countries, psychosocial interventions for pregnant adolescents and adolescent parents have resulted in small- to moderate-sized beneficial effects on adolescents’ positive mental health and school attendance. Though the conclusion related to positive mental health was based on very low quality evidence, the conclusion related to school attendance was based on high quality evidence. More high-quality evidence is urgently needed, especially from LMICs, to establish the effectiveness of psychosocial interventions on a range of additional interlinked mental health outcomes among pregnant and parenting adolescents.

## Supplementary information

**Additional file 1.**

**Additional file 2.**

**Additional file 3.**

**Additional file 4.**

## Data Availability

The datasets used and/or analysed during the current study are available from the corresponding author on reasonable request.
