# Supplementary material for: Psychosocial interventions targeting mental health in pregnant adolescents and adolescent parents: a systematic review
Source: Reprod Health. 2020 May 14;17:65. doi: 10.1186/s12978-020-00913-y (PMC7227359; doi:10.1186/s12978-020-00913-y)
Supplement: Supplementary file 1 — Additional file 1. [file 12978_2020_913_MOESM1_ESM.docx]

**Supplemental file 1: Full search strategy**

As detailed in our publication, our systematic review of psychosocial interventions for pregnant adolescents and adolescent parents was part of a larger set of systematic reviews conducted for the WHO Guidelines on Mental Health Promotive and Preventive Interventions for Adolescents. The searches for all eight systematic reviews were conducted together, to avoid duplication of work. This full search strategy shared represented the full list of items searched, from which the 19 final results for our specific systematic review described in this publication were drawn.

*Notes on search terms: For databases where indexing is thorough (i.e., PUBMED, Embase, and PSYCHInfo) terms will be search using field code for subject headings. For other databases (i.e., ASSIA and ERIC) terms will be searched using the “Title and Abstract” field code. Each database will have its own set of search strings.*

**Search #1 (PICO 1)**

**MEDLINE**

| 1 | (Adolescen* OR Juvenile* OR Student OR Pupil OR Learner OR Teen* OR Young adult OR Young people OR Youth OR Young wom* OR Young men OR Young man OR Pubescent).ab,ti,sh. |
| --- | --- |
| 2 | (Education OR Indicated OR Intervention OR Prevent* OR Program* OR Promot* OR Support OR Targeted OR Therap* OR Training OR Treatment  Universal OR Ccbt OR Cognitive Behav* OR CBT OR (Social ADJ2 emotional learning) OR Psychosocial OR Psychoeduc* OR Psycho-educ* OR Interpersonal OR Life Skill*).ab,ti,sh. |
| 3 | randomized controlled trial.pt. OR controlled clinical trial.pt. OR randomi#ed.ab. OR placebo.ab. OR clinical trials as topic.sh. OR randomly.ab. OR trial.ti |
| 4 | (Well-being OR Wellbeing OR Well being OR Life satisfaction OR Quality of life OR Positive psychology OR Self-concept OR self concept OR Self-esteem OR Self esteem OR Self control OR Self-control OR Self-efficacy OR Self efficacy).ab,ti,sh. OR (Mental function* OR Individual function* OR Social Function* OR Emotion* Function* OR Resilienc* OR Coping OR Problem Solv* OR Relationship skills OR Interpersonal skills OR Communicat* OR Emotion* regulat* OR Mental health).ab,ti,sh. OR (Depress* OR Anxiety OR Anxious OR Internali* OR Sad* OR Mood OR Affect* OR Dysphoria OR Emotion*).ab,ti,sh. OR (Self-harm OR Self harm OR Cutting OR Suicid*).ab,ti,sh.  OR  (Alcohol OR Intoxication OR drink* OR Drunk* OR Marijuana OR marihuana OR hashish OR THC OR Cannabis OR Opioid* OR Opiate* OR morphine OR heroin OR methadone OR diamorphine OR diacetylmorphine OR fentanyl OR stimulant* OR ecstasy OR MDMA OR methylenedioxymethamphetamine OR methamphetamine OR amphetamine OR cocaine OR Substance abuse OR Substance use* OR Substance dependence OR Substance addiction OR Tobacco OR Cigarette* OR Smoking OR Drug*).ab,ti,sh.  OR  (Aggress* OR Violen* OR Conflict OR Conduct OR Externali* OR Disruptive OR oppositional OR Impulse control OR Peer problems OR Antisocial* OR Anti-social OR Fight* OR Bully* OR Anger* OR Rage OR Arrest* OR Incarcerat* OR Criminal behav*).ab,ti,sh. OR (Condom* OR Safe sex OR Unsafe sex OR Protected sex OR Responsible sex OR Unprotected sex OR Safe intercourse OR Unsafe intercourse OR Protected intercourse OR Unprotected intercourse OR Multiple sexual partners OR Multiple partners OR Number of partners OR Early sexual debut OR Early sexual initiation OR Age at first sex* OR Early intercourse OR Early first sex OR Concurrent partners).ab,ti,sh. OR (School OR School enrol* OR School drop* OR School retention rate OR School days missed OR School attendance OR Intention to return).ab,ti,sh. |
| 5 | 1 AND 2 AND 3 AND 4 |

**EMBASE**

| 1 | (Adolescen* OR Juvenile* OR Student OR Pupil OR Learner OR Teen* OR Young adult OR Young people OR Youth OR Young wom* OR Young men OR Young man OR Pubescent).ab,ti,sh. |
| --- | --- |
| 2 | (Education OR Indicated OR Intervention OR Prevent* OR Program* OR Promot* OR Support OR Targeted OR Therap* OR Training OR Treatment  Universal OR Ccbt OR Cognitive Behav* OR CBT OR (Social ADJ2 emotional learning) OR Psychosocial OR Psychoeduc* OR Psycho-educ* OR Interpersonal OR life skill*).ab,ti,sh. |
| 3 | ((random$ OR placebo$ OR single blind$ OR double blind$ OR triple blind$).ti,ab. OR exp randomized controlled trial/) NOT ((random sampl$ OR random digit$ OR random effect$ OR random survey OR random regression).ti,ab. NOT exp randomized controlled trial/) |
| 4 | (Well-being OR Wellbeing OR Well being OR Life satisfaction OR Quality of life OR Positive psychology OR Self-concept OR self concept OR Self-esteem OR Self esteem OR Self control OR Self-control OR Self-efficacy OR Self efficacy).ab,ti,sh. OR (Mental function* OR Individual function* OR Social Function* OR Emotion* Function* OR Resilienc* OR Coping OR Problem Solv* OR Relationship skills OR Interpersonal skills OR Communicat* OR Emotion* regulat* OR Mental health).ab,ti,sh. OR (Depress* OR Anxiety OR Anxious OR Internali* OR Sad* OR Mood OR Affect* OR Dysphoria OR Emotion*).ab,ti,sh. OR (Self-harm OR Self harm OR Cutting OR Suicid*).ab,ti,sh.  OR  (Alcohol OR Intoxication OR drink* OR Drunk* OR Marijuana OR marihuana OR hashish OR THC OR Cannabis OR Opioid* OR Opiate* OR morphine OR heroin OR methadone OR diamorphine OR diacetylmorphine OR fentanyl OR stimulant* OR ecstasy OR MDMA OR methylenedioxymethamphetamine OR methamphetamine OR amphetamine OR cocaine OR Substance abuse OR Substance use* OR Substance dependence OR Substance addiction OR Tobacco OR Cigarette* OR Smoking OR Drug*).ab,ti,sh.  OR  (Aggress* OR Violen* OR Conflict OR Conduct OR Externali* OR Disruptive OR oppositional OR Impulse control OR Peer problems OR Antisocial* OR Anti-social OR Fight* OR Bully* OR Anger* OR Rage OR Arrest* OR Incarcerat* OR Criminal behav*).ab,ti,sh. OR (Condom* OR Safe sex OR Unsafe sex OR Protected sex OR Responsible sex OR Unprotected sex OR Safe intercourse OR Unsafe intercourse OR Protected intercourse OR Unprotected intercourse OR Multiple sexual partners OR Multiple partners OR Number of partners OR Early sexual debut OR Early sexual initiation OR Age at first sex* OR Early intercourse OR Early first sex OR Concurrent partners).ab,ti,sh. OR (School OR School enrol* OR School drop* OR School retention rate OR School days missed OR School attendance OR Intention to return).ab,ti,sh. |
| 5 | 1 AND 2 AND 3 AND 4 |

**PsycINFO**

| 1 | (Adolescen* OR Juvenile* OR Student OR Pupil OR Learner OR Teen* OR Young adult OR Young people OR Youth OR Young wom* OR Young men OR Young man OR Pubescent).ti,ab,sh |
| --- | --- |
| 2 | (Education OR Indicated OR Intervention OR Prevent* OR Program* OR Promot* OR Support OR Targeted OR Therap* OR Training OR Treatment  Universal OR Ccbt OR Cognitive Behav* OR CBT OR Social ADJ2 emotional learning OR Psychosocial OR Psychoeduc* OR Psycho-educ* OR Interpersonal OR life skill*).ti,ab,sh |
| 3 | "Treatment Effectiveness Evaluation"/ OR exp Treatment Outcomes/ OR "Placebo"/ OR "Followup Studies"/ OR (placebo* OR random* OR "comparative stud*" OR (clinical adj3 trial*) OR (research adj3 design) OR (evaluat* adj3 stud*) OR (prospectiv* adj3 stud*) OR ((singl* OR doubl* OR trebl* OR tripl*) adj3 (blind* OR mask*))).ab,ti. |
| 4 | (Well-being OR Wellbeing OR Well being OR Life satisfaction OR Quality of life OR Positive psychology OR Self-concept OR self concept OR Self-esteem OR Self esteem OR Self control OR Self-control OR Self-efficacy OR Self efficacy).ti,ab,sh  OR  (Mental function* OR Individual function* OR Social Function* OR Emotion* Function* OR Resilienc* OR Coping OR Problem Solv* OR Relationship skills OR Interpersonal skills OR Communicat* OR Emotion* regulat* OR Mental health).ti,ab,sh  OR  (Depress* OR Anxiety OR Anxious OR Internali* OR Sad* OR Mood OR Affect* OR Dysphoria OR Emotion*).ti,ab,sh  OR  (Self-harm OR Self harm OR Cutting OR Suicid*).ti,ab,sh  OR  (Alcohol OR Intoxication OR drink* OR Drunk* OR Marijuana OR marihuana OR hashish OR THC OR Cannabis OR Opioid* OR Opiate* OR morphine OR heroin OR methadone OR diamorphine OR diacetylmorphine OR fentanyl OR stimulant* OR ecstasy OR MDMA OR methylenedioxymethamphetamine OR methamphetamine OR amphetamine OR cocaine).ti,ab,sh OR (Substance abuse OR Substance use* OR Substance dependence OR Substance addiction OR Tobacco OR Cigarette* OR Smoking OR Drug*).ti,ab,sh  OR  (Aggress* OR Violen* OR Conflict OR Conduct OR Externali* OR Disruptive OR oppositional OR Impulse control OR Peer problems OR Antisocial* OR Anti-social OR Fight* OR Bully* OR Anger* OR Rage OR Arrest* OR Incarcerat* OR Criminal behav*).ti,ab,sh  OR  (Condom* OR Safe sex OR Unsafe sex OR Protected sex OR Responsible sex OR Unprotected sex OR Safe intercourse OR Unsafe intercourse OR Protected intercourse OR Unprotected intercourse OR Multiple sexual partners OR Multiple partners OR Number of partners OR Early sexual debut OR Early sexual initiation OR Age at first sex* OR Early intercourse OR Early first sex OR Concurrent partners).ti,ab,sh  OR  (School OR School enrol* School drop* OR School retention rate OR School days missed OR School attendance OR Intention to return).ti,ab,sh |
| 5 | 1 AND 2 AND 3 AND 4 |

**ASSIA (ProQuest)**

| 1 | TI,AB(Adolescen* OR Juvenile* OR Student OR Pupil OR Learner OR Teen* OR Young adult OR Young people OR Youth OR Young wom* OR Young men OR Young man OR Pubescent) |
| --- | --- |
| 2 | TI,AB (Education OR Indicated OR Intervention OR Prevent* OR Program* OR Promot* OR Support OR Targeted OR Therap* OR Training OR Treatment OR Universal OR Ccbt OR Cognitive Behav* OR CBT OR (Social NEAR/2 emotional learning) OR Psychosocial OR Psychoeduc* OR Psycho-educ* OR Interpersonal OR life skill*) |
| 3 | TI,AB (RCT OR Randomi* OR Randomly OR Trial OR Groups OR Crossover OR Cross over OR Cross-over OR Double blind OR Factorial) |
| 4 | TI,AB (Well-being OR Wellbeing OR Well being OR Life satisfaction OR Quality of life OR Positive psychology OR Self-concept OR self concept OR Self-esteem OR Self esteem OR Self control OR Self-control OR Self-efficacy OR Self efficacy) OR TI,AB (Mental function* OR Individual function* OR Social Function* OR Emotion* Function* OR Resilienc* OR Coping OR Problem Solv* OR Relationship skills OR Interpersonal skills OR Communicat* OR Emotion* regulat* OR Mental health) OR TI,AB (Depress* OR Anxiety OR Anxious OR Internali* OR Sad* OR Mood OR Affect* OR Dysphoria OR Emotion*) OR TI,AB (Self-harm OR Self harm OR Cutting OR Suicid*) OR TI,AB (Alcohol OR Intoxication OR drink* OR Drunk* OR Marijuana OR marihuana OR hashish OR THC OR Cannabis OR Opioid* OR Opiate* OR morphine OR heroin OR methadone OR diamorphine OR diacetylmorphine OR fentanyl OR stimulant* OR ecstasy OR MDMA OR methylenedioxymethamphetamine OR methamphetamine OR amphetamine OR cocaine OR Substance abuse OR Substance use OR Substance dependence OR Substance addiction OR Tobacco OR Cigarette* OR Smoking OR Drug*) OR TI,AB (Aggress* OR Violen* OR Conflict OR Conduct OR Externali* OR Disruptive OR oppositional OR Impulse control OR Peer problems OR Antisocial* OR Anti-social OR Fight* OR Bully* OR Anger* OR Rage OR Arrest* OR Incarcerat* OR Criminal behav*) OR TI,AB (Condom* OR Safe sex OR Unsafe sex OR Protected sex OR Responsible sex OR Unprotected sex OR Safe intercourse OR Unsafe intercourse OR Protected intercourse OR Unprotected intercourse OR Multiple sexual partners OR Multiple partners OR Number of partners OR Early sexual debut OR Early sexual initiation OR Age at first sex* OR Early intercourse OR Early first sex OR Concurrent partners) OR TI,AB (School OR School enrol* OR School drop* OR School retention rate OR School days missed OR School attendance OR Intention to return) |
| 5 | 1 AND 2 AND 3 AND 4 |

**ERIC**

| 1 | AB(Adolescen* OR Juvenile* OR Student OR Pupil OR Learner OR Teen* OR Young adult OR Young people OR Youth OR Young wom* OR Young men OR Young man OR Pubescent) OR TI(Adolescen* OR Juvenile* OR Student OR Pupil OR Learner OR Teen* OR Young adult OR Young people OR Youth OR Young wom* OR Young men OR Young man OR Pubescent) |
| --- | --- |
| 2 | AB(Education OR Indicated OR Intervention OR Prevent* OR Program* OR Promot* OR Support OR Targeted OR Therap* OR Training OR Treatment  Universal OR Ccbt OR Cognitive Behav* OR CBT OR (Social N2 emotional learning) OR Psychosocial OR Psychoeduc* OR Psycho-educ* OR Interpersonal OR life skill*) OR  TI(Education OR Indicated OR Intervention OR Prevent* OR Program* OR Promot* OR Support OR Targeted OR Therap* OR Training OR Treatment  Universal OR Ccbt OR Cognitive Behav* OR CBT OR (Social N2 emotional learning) OR Psychosocial OR Psychoeduc* OR Psycho-educ* OR Interpersonal OR life skill*) |
| 3 | AB(Randomi* OR Randomly OR RCT OR Trial OR Arms OR Double blind OR double-blind OR Factorial OR "control group" OR "controlled trial") OR TI(Randomi* OR Randomly OR RCT OR Trial OR Arms OR Double blind OR double-blind OR Factorial OR "control group" OR "controlled trial") |
| 4 | AB(Well-being OR Wellbeing OR Well being OR Life satisfaction OR Quality of life OR Positive psychology OR Self-concept OR self concept OR Self-esteem OR Self esteem OR Self control OR Self-control OR Self-efficacy OR Self efficacy) OR TI(Well-being OR Wellbeing OR Well being OR Life satisfaction OR Quality of life OR Positive psychology OR Self-concept OR self concept OR Self-esteem OR Self esteem OR Self control OR Self-control OR Self-efficacy OR Self efficacy) OR AB(Mental function* OR Individual function* OR Social Function* OR Emotion* Function* OR Resilienc* OR Coping OR Problem Solv* OR Relationship skills OR Interpersonal skills OR Communicat* OR Emotion* regulat* OR Mental health) OR TI(Mental function* OR Individual function* OR Social Function* OR Emotion* Function* OR Resilienc* OR Coping OR Problem Solv* OR Relationship skills OR Interpersonal skills OR Communicat* OR Emotion* regulat* OR Mental health) OR AB(Depress* OR Anxiety OR Anxious OR Internali* OR Sad* OR Mood OR Affect* OR Dysphoria OR Emotion*) OR TI(Depress* OR Anxiety OR Anxious OR Internali* OR Sad* OR Mood OR Affect* OR Dysphoria OR Emotion*) OR AB(Self-harm OR Self harm OR Cutting OR Suicid*) OR TI(Self-harm OR Self harm OR Cutting OR Suicid*) OR AB(Alcohol OR Intoxication OR drink* OR Drunk* OR Marijuana OR marihuana OR hashish OR THC OR Cannabis OR Opioid* OR Opiate* OR morphine OR heroin OR methadone OR diamorphine OR diacetylmorphine OR fentanyl OR stimulant* OR ecstasy OR MDMA OR methylenedioxymethamphetamine OR methamphetamine OR amphetamine OR cocaine OR Substance abuse OR Substance use OR Substance dependence OR Substance addiction OR Tobacco OR Cigarette* OR Smoking or Drug*) OR TI(Alcohol OR Intoxication OR drink* OR Drunk* OR Marijuana OR marihuana OR hashish OR THC OR Cannabis OR Opioid* OR Opiate* OR morphine OR heroin OR methadone OR diamorphine OR diacetylmorphine OR fentanyl OR stimulant* OR ecstasy OR MDMA OR methylenedioxymethamphetamine OR methamphetamine OR amphetamine OR cocaine OR Substance abuse OR Substance use OR Substance dependence OR Substance addiction OR Tobacco OR Cigarette* OR Smoking OR Drug*) OR AB(Aggress* OR Violen* OR Conflict OR Conduct OR Externali* OR Disruptive OR oppositional OR Impulse control OR Peer problems OR Antisocial* OR Anti-social OR Fight* OR Bully* OR Anger* OR Rage OR Arrest* OR Incarcerat* OR Criminal behav*) OR TI(Aggress* OR Violen* OR Conflict OR Conduct OR Externali* OR Disruptive OR oppositional OR Impulse control OR Peer problems OR Antisocial* OR Anti-social OR Fight* OR Bully* OR Anger* OR Rage OR Arrest* OR Incarcerat* OR Criminal behav*) OR AB(Condom* OR Safe sex OR Unsafe sex OR Protected sex OR Responsible sex OR Unprotected sex OR Safe intercourse OR Unsafe intercourse OR Protected intercourse OR Unprotected intercourse OR Multiple sexual partners OR Multiple partners OR Number of partners OR Early sexual debut OR Early sexual initiation OR Age at first sex* OR Early intercourse OR Early first sex OR Concurrent partners) OR TI(Condom* OR Safe sex OR Unsafe sex OR Protected sex OR Responsible sex OR Unprotected sex OR Safe intercourse OR Unsafe intercourse OR Protected intercourse OR Unprotected intercourse OR Multiple sexual partners OR Multiple partners OR Number of partners OR Early sexual debut OR Early sexual initiation OR Age at first sex* OR Early intercourse OR Early first sex OR Concurrent partners) OR AB(School OR School enrol* OR School drop* OR School retention rate OR School days missed OR School attendance OR Intention to return) OR TI(School OR School enrol* OR School drop* OR School retention rate OR School days missed OR School attendance OR Intention to return) |
| 5 | S1 AND S2 AND S3 AND S4 |

**Search #2 (PICO 2-6)**

Search 🡪 adolescents + targeted / indicated (spaced with OR) + intervention + RCT filter + outcomes

**PUBMED**

| 1 | (Adolescen* OR Juvenile* OR Student OR Pupil OR Learner OR Teen* OR Young adult OR Young people OR Youth OR Young wom* OR Young men OR Young man OR Pubescent).ab,ti,sh. |
| --- | --- |
| 2 | (Violen* OR Conflict).ab,ti,sh. |
| 3 | (Poverty OR Food insecur* OR Asset poor OR Asset index OR Disadvantaged OR Homeless OR Indigent OR Low income OR Low-income OR Poor OR Breadline OR Depriv* OR Extreme poverty OR Impoverished OR Destitute).ab,ti,sh. |
| 4 | (Refugee OR Forced migrant OR Displace* OR Migrant OR Asylum seeker OR Persecution OR Civil disruption OR Conflict OR Humanitarian OR Political unrest OR Emergency OR Natural Disaster OR Fire OR Wildfire OR Hurricane OR Tornado OR Flood OR Tsunami OR Monsoon OR Earthquake OR Volcano OR Sandstorm OR Storm OR Landslide OR Mudslide OR Heatwave OR Cyclone OR Blizzard OR Tropical storm OR Drought OR Famine OR War).ab,ti,sh. |
| 5 | (Perinatal OR Prenatal OR Postnatal OR Antenatal OR Peri-natal OR Pre-natal OR Post-natal OR Ante-natal OR Mother OR Father OR Parent* OR Expecting OR Expectant OR Pregnan* OR Maternal OR Paternal).ab,ti,sh. |
| 6 | (HIV OR AIDS).ab,ti,sh. |
| 7 | (Depress* OR Anxiety OR Anxious OR Internali* OR Sad* OR Mood OR Affect* OR Dysphoria OR Emotion*).ab,ti,sh. |
| 8 | (Aggress* OR Violen* OR Conflict OR Conduct OR Externali* OR Disruptive OR oppositional OR Impulse control OR Peer problems OR Antisocial* OR Anti-social OR Fight* OR Bully* OR Anger* OR Rage).ab,ti,sh. |
| 9 | (Education OR Indicated OR Intervention OR Prevent* OR Program* OR Promot* OR Support OR Targeted OR Therap* OR Training OR Treatment OR Universal OR Ccbt OR Cognitive Behav* OR CBT OR (Social ADJ2 emotional learning) OR Psychosocial OR Psychoeduc* OR Psycho-educ* OR Interpersonal OR Life skill*).ab,ti,sh. |
| 10 | randomized controlled trial.pt. OR controlled clinical trial.pt. OR randomi#ed.ab. OR placebo.ab. OR clinical trials as topic.sh. OR randomly.ab. OR trial.ti |
| 11 | (Well-being OR Wellbeing OR Well being OR Life satisfaction OR Quality of life OR Positive psychology OR Self-concept OR self concept OR Self-esteem OR Self esteem OR Self control OR Self-control OR Self-efficacy OR Self efficacy).ab,ti,sh. OR (Mental function* OR Individual function* OR Social Function* OR Emotion* Function* OR Resilienc* OR Coping OR Problem Solv* OR Relationship skills OR Interpersonal skills OR Communicat* OR Emotion* regulat* OR Mental health).ab,ti,sh.  OR  (Depress* OR Anxiety OR Anxious OR Internali* OR Sad* OR Mood OR Affect* OR Dysphoria OR Emotion*).ab,ti,sh. OR (Conduct OR Oppositional behav* OR Oppositional defiant OR ODD).ab,ti,sh. OR (Self-harm OR Self harm OR Cutting OR Suicid*).ab,ti,sh.  OR  (Alcohol OR Intoxication OR drink* OR Drunk* OR Marijuana OR marihuana OR hashish OR THC OR Cannabis OR Opioid* OR Opiate* OR morphine OR heroin OR methadone OR diamorphine OR diacetylmorphine OR fentanyl OR stimulant* OR ecstasy OR MDMA OR methylenedioxymethamphetamine OR methamphetamine OR amphetamine OR cocaine OR Substance abuse OR Substance use* OR Substance dependence OR Substance addiction OR Tobacco OR Cigarette* OR Smoking OR Drug*).ti,ab,sh  OR  (Aggress* OR Violen* OR Conflict OR Conduct OR Externali* OR Disruptive OR oppositional OR Impulse control OR Peer problems OR Antisocial* OR Anti-social OR Fight* OR Bully* OR Anger* OR Rage OR Arrest* OR Incarcerat* OR Criminal behav*).ti,ab,sh OR (Condom* OR Safe sex OR Unsafe sex OR Protected sex OR Responsible sex OR Unprotected sex OR Safe intercourse OR Unsafe intercourse OR Protected intercourse OR Unprotected intercourse OR Multiple sexual partners OR Multiple partners OR Number of partners OR Early sexual debut OR Early sexual initiation OR Age at first sex* OR Early intercourse OR Early first sex OR Concurrent partners).ti,ab,sh OR (School OR School enrol* OR School drop* OR School retention rate OR School days missed OR School attendance OR Intention to return to school).ti,ab,sh  OR  (Antenatal OR Initiation of antenatal care OR Antenatal visit* OR Number of antenatal visits OR Postnatal visit* OR Post-delivery visit* OR Immuniz* OR Immunis* OR Vaccinat* OR Contracepti* OR Birth control OR Family planning OR Fertility control).ti,ab,sh OR (Child development OR Positive parenting OR Parenting OR Parenting stress OR Parenting self-efficacy OR Discipline).ti,ab,sh OR (Parenting behav* OR Responsive parenting OR Sensitiv* OR Sensitive parenting OR (Positive interactions ADJ2 child) OR Discipline OR Behav* management).ti,ab,sh OR (Victimi* OR Relationship conflict OR Relationship violence OR Dating violence OR Coercive sex OR Gender-based violence OR GBV OR Intimate partner violence OR Domestic violence OR Domestic abuse OR Relationship abuse OR Verbal abuse OR Psychological abuse OR Physical abuse).ti,ab,sh OR (Adher* OR ARV initiation OR ARV discontinuation OR ARV OR ART OR Antiretroviral Treatment OR ARV adherence OR Viral load OR Viral suppression OR CD4 OR Medication possession ratio OR Pill count OR Tablet count OR Electronic monitoring of adherence).ti,ab,sh |
| 12 | (Stress disorder OR Stress OR PTSD OR Post traumatic stress disorder OR Post-traumatic stress disorder OR Post-trauma* OR Post trauma* OR Psychological trauma OR Trauma* OR Complex PTSD).ti,ab,sh |
| 13 | (1 AND (2 OR 3 OR 4 OR 5 OR 6 OR 7 OR 8) AND 9 AND 10 AND 11) OR (1 AND (2 OR 3 OR 4) AND 9 AND 10 AND 11 AND 12) |

**EMBASE**

| 1 | (Adolescen* OR Juvenile* OR Student OR Pupil OR Learner OR Teen* OR Young adult OR Young people OR Youth OR Young wom* OR Young men OR Young man OR Pubescent).ab,ti,sh. |
| --- | --- |
| 2 | (Violen* OR Conflict).ab,ti,sh. |
| 3 | (Poverty OR Food insecur* OR Asset poor OR Asset index OR Disadvantaged OR Homeless OR Indigent OR Low income OR Low-income OR Poor OR Breadline OR Depriv* OR Extreme poverty OR Impoverished OR Destitute).ab,ti,sh. |
| 4 | (Refugee OR Forced migrant OR Displace* OR Migrant OR Asylum seeker OR Persecution OR Civil disruption OR Conflict OR Humanitarian OR Political unrest OR Emergency OR Natural Disaster OR Fire OR Wildfire OR Hurricane OR Tornado OR Flood OR Tsunami OR Monsoon OR Earthquake OR Volcano OR Sandstorm OR Storm OR Landslide OR Mudslide OR Heatwave OR Cyclone OR Blizzard OR Tropical storm OR Drought OR Famine OR War).ab,ti,sh. |
| 5 | (Perinatal OR Prenatal OR Postnatal OR Antenatal OR Peri-natal OR Pre-natal OR Post-natal OR Ante-natal OR Mother OR Father OR Parent* OR Expecting OR Expectant OR Pregnan* OR Maternal OR Paternal).ab,ti,sh. |
| 6 | (HIV OR AIDS).ab,ti,sh. |
| 7 | (Depress* OR Anxiety OR Anxious OR Internali* OR Sad* OR Mood OR Affect* OR Dysphoria OR Emotion*).ab,ti,sh. |
| 8 | (Aggress* OR Violen* OR Conflict OR Conduct OR Externali* OR Disruptive OR oppositional OR Impulse control OR Peer problems OR Antisocial* OR Anti-social OR Fight* OR Bully* OR Anger* OR Rage).ab,ti,sh. |
| 9 | (Education OR Indicated OR Intervention OR Prevent* OR Program* OR Promot* OR Support OR Targeted OR Therap* OR Training OR Treatment OR Universal OR Ccbt OR Cognitive Behav* OR CBT OR (Social ADJ2 emotional learning) OR Psychosocial OR Psychoeduc* OR Psycho-educ* OR Interpersonal OR Life Skill*).ab,ti,sh. |
| 10 | ((random$ OR placebo$ OR single blind$ OR double blind$ OR triple blind$).ti,ab. OR exp randomized controlled trial/) NOT ((random sampl$ OR random digit$ OR random effect$ OR random survey OR random regression).ti,ab. NOT exp randomized controlled trial/) |
| 11 | (Well-being OR Wellbeing OR Well being OR Life satisfaction OR Quality of life OR Positive psychology OR Self-concept OR self concept OR Self-esteem OR Self esteem OR Self control OR Self-control OR Self-efficacy OR Self efficacy).ab,ti,sh. OR (Mental function* OR Individual function* OR Social Function* OR Emotion* Function* OR Resilienc* OR Coping OR Problem Solv* OR Relationship skills OR Interpersonal skills OR Communicat* OR Emotion* regulat* OR Mental health).ab,ti,sh. OR (Depress* OR Anxiety OR Anxious OR Internali* OR Sad* OR Mood OR Affect* OR Dysphoria OR Emotion*).ab,ti,sh. OR (Conduct OR Oppositional behav* OR Oppositional defiant OR ODD).ab,ti,sh. OR (Self-harm OR Self harm OR Cutting OR Suicid*).ab,ti,sh.  OR  (Alcohol OR Intoxication OR drink* OR Drunk* OR Marijuana OR marihuana OR hashish OR THC OR Cannabis OR Opioid* OR Opiate* OR morphine OR heroin OR methadone OR diamorphine OR diacetylmorphine OR fentanyl OR stimulant* OR ecstasy OR MDMA OR methylenedioxymethamphetamine OR methamphetamine OR amphetamine OR cocaine OR Substance abuse OR Substance use* OR Substance dependence OR Substance addiction OR Tobacco OR Cigarette* OR Smoking OR Drug*).ab,ti,sh.  OR  (Aggress* OR Violen* OR Conflict OR Conduct OR Externali* OR Disruptive OR oppositional OR Impulse control OR Peer problems OR Antisocial* OR Anti-social OR Fight* OR Bully* OR Anger* OR Rage OR Arrest* OR Incarcerat* OR Criminal behav*).ab,ti,sh. OR (Condom* OR Safe sex OR Unsafe sex OR Protected sex OR Responsible sex OR Unprotected sex OR Safe intercourse OR Unsafe intercourse OR Protected intercourse OR Unprotected intercourse OR Multiple sexual partners OR Multiple partners OR Number of partners OR Early sexual debut OR Early sexual initiation OR Age at first sex* OR Early intercourse OR Early first sex OR Concurrent partners).ab,ti,sh. OR (School OR School enrol* OR School drop* OR School retention rate OR School days missed OR School attendance OR Intention to return).ab,ti,sh.  OR  (Antenatal OR Initiation of antenatal care OR Antenatal visit* OR Number of antenatal visits OR Postnatal visit* OR Post-delivery visit* OR Immuniz* OR Immunis* OR Vaccinat* OR Contracepti* OR Birth control OR Family planning OR Fertility control).ti,ab,sh OR (Child development OR Positive parenting OR Parenting OR Parenting stress OR Parenting self-efficacy OR Discipline).ti,ab,sh OR (Parenting behav* OR Responsive parenting OR Sensitiv* OR Sensitive parenting OR (Positive interactions ADJ2 child) OR Discipline OR Behav* management).ti,ab,sh OR (Victimi* OR Relationship conflict OR Relationship violence OR Dating violence OR Coercive sex OR Gender-based violence OR GBV OR Intimate partner violence OR Domestic violence OR Domestic abuse OR Relationship abuse OR Verbal abuse OR Psychological abuse OR Physical abuse).ti,ab,sh OR (Adher* OR ARV initiation OR ARV discontinuation OR ARV OR ART OR Antiretroviral Treatment OR ARV adherence OR Viral load OR Viral suppression OR CD4 OR Medication possession ratio OR Pill count OR Tablet count OR Electronic monitoring of adherence).ti,ab,sh |
| 12 | (Stress disorder OR Stress OR PTSD OR Post traumatic stress disorder OR Post-traumatic stress disorder OR Post-trauma* OR Post trauma* OR Psychological trauma OR Trauma* OR Complex PTSD).ti,ab,sh |
| 13 | (1 AND (2 OR 3 OR 4 OR 5 OR 6 OR 7 OR 8) AND 9 AND 10 AND 11) OR (1 AND (2 OR 3 OR 4) AND 9 AND 10 AND 11 AND 12) |

**PsycINFO**

| 1 | (Adolescen* OR Juvenile* OR Student OR Pupil OR Learner OR Teen* OR Young adult OR Young people OR Youth OR Young wom* OR Young men OR Young man OR Pubescent).ti,ab,sh |
| --- | --- |
| 2 | (Violen* OR Conflict).ti,ab,sh |
| 3 | (Poverty OR Food insecur* OR Asset poor OR Asset index OR Disadvantaged OR Homeless OR Indigent OR Low income OR Low-income OR Poor OR Breadline OR Depriv* OR Extreme poverty OR Impoverished OR Destitute).ti,ab,sh |
| 4 | (Refugee OR Forced migrant OR Displace* OR Migrant OR Asylum seeker OR Persecution OR Civil disruption OR Conflict OR Humanitarian OR Political unrest OR Emergency OR Natural Disaster OR Fire OR Wildfire OR Hurricane OR Tornado OR Flood OR Tsunami OR Monsoon OR Earthquake OR Volcano OR Sandstorm OR Storm OR Landslide OR Mudslide OR Heatwave OR Cyclone OR Blizzard OR Tropical storm OR Drought OR Famine OR War).ti,ab,sh |
| 5 | (Perinatal OR Prenatal OR Postnatal OR Antenatal OR Peri-natal OR Pre-natal OR Post-natal OR Ante-natal OR Mother OR Father OR Parent* OR Expecting OR Expectant OR Pregnan* OR Maternal OR Paternal).ti,ab,sh |
| 6 | (HIV OR AIDS).ti,ab,sh |
| 7 | (Depress* OR Anxiety OR Anxious OR Internali* OR Sad* OR Mood OR Affect* OR Dysphoria OR Emotion*).ti,ab,sh |
| 8 | (Aggress* OR Violen* OR Conflict OR Conduct OR Externali* OR Disruptive OR oppositional OR Impulse control OR Peer problems OR Antisocial* OR Anti-social OR Fight* OR Bully* OR Anger* OR Rage).ti,ab,sh |
| 9 | (Education OR Indicated OR Intervention OR Prevent* OR Program* OR Promot* OR Support OR Targeted OR Therap* OR Training OR Treatment OR Universal OR Ccbt OR Cognitive Behav* OR CBT OR (Social ADJ2 emotional learning) OR Psychosocial OR Psychoeduc* OR Psycho-educ* OR Interpersonal OR Life Skill*).ti,ab,sh |
| 10 | "Treatment Effectiveness Evaluation"/ OR exp Treatment Outcomes/ OR "Placebo"/ OR "Followup Studies"/ OR (placebo* OR random* OR "comparative stud*" OR (clinical adj3 trial*) OR (research adj3 design) OR (evaluat* adj3 stud*) OR (prospectiv* adj3 stud*) OR ((singl* OR doubl* OR trebl* OR tripl*) adj3 (blind* OR mask*))).ab,ti. |
| 11 | (Well-being OR Wellbeing OR Well being OR Life satisfaction OR Quality of life OR Positive psychology OR Self-concept OR self concept OR Self-esteem OR Self esteem OR Self control OR Self-control OR Self-efficacy OR Self efficacy).ti,ab,sh  OR  (Mental function* OR Individual function* OR Social Function* OR Emotion* Function* OR Resilienc* OR Coping OR Problem Solv* OR Relationship skills OR Interpersonal skills OR Communicat* OR Emotion* regulat* OR Mental health).ti,ab,sh  OR  (Depress* OR Anxiety OR Anxious OR Internali* OR Sad* OR Mood OR Affect* OR Dysphoria OR Emotion*).ti,ab,sh  OR  (Conduct OR Oppositional behav* OR Oppositional defiant OR ODD).ab,ti,sh  OR  (Self-harm OR Self harm OR Cutting OR Suicid*).ti,ab,sh  OR  (Alcohol OR Intoxication OR drink* OR Drunk* OR Marijuana OR marihuana OR hashish OR THC OR Cannabis OR Opioid* OR Opiate* OR morphine OR heroin OR methadone OR diamorphine OR diacetylmorphine OR fentanyl OR stimulant* OR ecstasy OR MDMA OR methylenedioxymethamphetamine OR methamphetamine OR amphetamine OR cocaine OR Substance abuse OR Substance use* OR Substance dependence OR Substance addiction OR Tobacco OR Cigarette* OR Smoking OR Drug*).ti,ab,sh  OR  (Aggress* OR Violen* OR Conflict OR Conduct OR Externali* OR Disruptive OR oppositional OR Impulse control OR Peer problems OR Antisocial* OR Anti-social OR Fight* OR Bully* OR Anger* OR Rage OR Arrest* OR Incarcerat* OR Criminal behav*).ti,ab,sh  OR  (Condom* OR Safe sex OR Unsafe sex OR Protected sex OR Responsible sex OR Unprotected sex OR Safe intercourse OR Unsafe intercourse OR Protected intercourse OR Unprotected intercourse OR Multiple sexual partners OR Multiple partners OR Number of partners OR Early sexual debut OR Early sexual initiation OR Age at first sex* OR Early intercourse OR Early first sex OR Concurrent partners).ti,ab,sh OR (School OR School enrol* OR School drop* OR School retention rate OR School days missed OR School attendance OR Intention to return to school).ti,ab,sh  OR  (Antenatal OR Initiation of antenatal care OR Antenatal visit* OR Number of antenatal visits OR Postnatal visit* OR Post-delivery visit* OR Immuniz* OR Immunis* OR Vaccinat* OR Contracepti* OR Birth control OR Family planning OR Fertility control).ti,ab,sh OR (Child development OR Positive parenting OR Parenting OR Parenting stress OR Parenting self-efficacy OR Discipline).ti,ab,sh OR (Parenting behav* OR Responsive parenting OR Sensitiv* OR Sensitive parenting OR (Positive interactions ADJ2 child) OR Discipline OR Behav* management).ti,ab,sh OR (Victimi* OR Relationship conflict OR Relationship violence OR Dating violence OR Coercive sex OR Gender-based violence OR GBV OR Intimate partner violence OR Domestic violence OR Domestic abuse OR Relationship abuse OR Verbal abuse OR Psychological abuse OR Physical abuse).ti,ab,sh OR (Adher* OR ARV initiation OR ARV discontinuation OR ARV OR ART OR Antiretroviral Treatment OR ARV adherence OR Viral load OR Viral suppression OR CD4 OR Medication possession ratio OR Pill count OR Tablet count OR Electronic monitoring of adherence).ti,ab,sh |
| 12 | (Stress disorder OR Stress OR PTSD OR Post traumatic stress disorder OR Post-traumatic stress disorder OR Post-trauma* OR Post trauma* OR Psychological trauma OR Trauma* OR Complex PTSD).ti,ab,sh |
| 13 | (1 AND (2 OR 3 OR 4 OR 5 OR 6 OR 7 OR 8) AND 9 AND 10 AND 11) OR (1 AND (2 OR 3 OR 4) AND 9 AND 10 AND 11 AND 12) |

**ASSIA (ProQuest)**

| 1 | TI,AB(Adolescen* OR Juvenile* OR Student OR Pupil OR Learner OR Teen* OR Young adult OR Young people OR Youth OR Young wom* OR Young men OR Young man OR Pubescent) |
| --- | --- |
| 2 | TI,AB(Violen* OR Conflict) |
| 3 | TI,AB(Poverty OR Food insecur* OR Asset poor OR Asset index OR Disadvantaged OR Homeless OR Indigent OR Low income OR Low-income OR Poor OR Breadline OR Depriv* OR Extreme poverty OR Impoverished OR Destitute) |
| 4 | TI,AB(Refugee OR Forced migrant OR Displace* OR Migrant OR Asylum seeker OR Persecution OR Civil disruption OR Conflict OR Humanitarian OR Political unrest OR Emergency OR Natural Disaster OR Fire OR Wildfire OR Hurricane OR Tornado OR Flood OR Tsunami OR Monsoon OR Earthquake OR Volcano OR Sandstorm OR Storm OR Landslide OR Mudslide OR Heatwave OR Cyclone OR Blizzard OR Tropical storm OR Drought OR Famine OR War) |
| 5 | TI,AB(Perinatal OR Prenatal OR Postnatal OR Antenatal OR Peri-natal OR Pre-natal OR Post-natal OR Ante-natal OR Mother OR Father OR Parent* OR Expecting OR Expectant OR Pregnan* OR Maternal OR Paternal) |
| 6 | TI,AB(HIV OR AIDS) |
| 7 | TI,AB(Depress* OR Anxiety OR Anxious OR Internali* OR Sad* OR Mood OR Affect* OR Dysphoria OR Emotion*) |
| 8 | TI,AB(Aggress* OR Violen* OR Conflict OR Conduct OR Externali* OR Disruptive OR oppositional OR Impulse control OR Peer problems OR Antisocial* OR Anti-social OR Fight* OR Bully* OR Anger* OR Rage) |
| 9 | TI,AB (Education OR Indicated OR Intervention OR Prevent* OR Program* OR Promot* OR Support OR Targeted OR Therap* OR Training OR Treatment OR Universal OR Ccbt OR Cognitive Behav* OR CBT OR (Social NEAR/2 emotional learning) OR Psychosocial OR Psychoeduc* OR Psycho-educ* OR Interpersonal OR life skill*) |
| 10 | TI,AB (RCT OR Randomi* OR Randomly OR Trial OR Groups OR Crossover OR Cross over OR Cross-over OR Double blind OR Factorial) |
| 11 | TI,AB (Well-being OR Wellbeing OR Well being OR Life satisfaction OR Quality of life OR Positive psychology OR Self-concept OR self concept OR Self-esteem OR Self esteem OR Self control OR Self-control OR Self-efficacy OR Self efficacy) OR TI,AB (Mental function* OR Individual function* OR Social Function* OR Emotion* Function* OR Resilienc* OR Coping OR Problem Solv* OR Relationship skills OR Interpersonal skills OR Communicat* OR Emotion* regulat* OR Mental health) OR TI,AB (Depress* OR Anxiety OR Anxious OR Internali* OR Sad* OR Mood OR Affect* OR Dysphoria OR Emotion*) OR TI,AB (Conduct OR Oppositional behav* OR Oppositional defiant OR ODD) OR TI,AB (Self-harm OR Self harm OR Cutting OR Suicid*) OR TI,AB (Alcohol OR Intoxication OR drink* OR Drunk* OR Marijuana OR marihuana OR hashish OR THC OR Cannabis OR Opioid* OR Opiate* OR morphine OR heroin OR methadone OR diamorphine OR diacetylmorphine OR fentanyl OR stimulant* OR ecstasy OR MDMA OR methylenedioxymethamphetamine OR methamphetamine OR amphetamine OR cocaine OR Substance abuse OR Substance use OR Substance dependence OR Substance addiction OR Tobacco OR Cigarette* OR Smoking OR Drug*) OR TI,AB (Aggress* OR Violen* OR Conflict OR Conduct OR Externali* OR Disruptive OR oppositional OR Impulse control OR Peer problems OR Antisocial* OR Anti-social OR Fight* OR Bully* OR Anger* OR Rage OR Arrest* OR Incarcerat* OR Criminal behav*) OR TI,AB (Condom* OR Safe sex OR Unsafe sex OR Protected sex OR Responsible sex OR Unprotected sex OR Safe intercourse OR Unsafe intercourse OR Protected intercourse OR Unprotected intercourse OR Multiple sexual partners OR Multiple partners OR Number of partners OR Early sexual debut OR Early sexual initiation OR Age at first sex* OR Early intercourse OR Early first sex OR Concurrent partners) OR TI,AB (School OR School enrol* OR School drop* OR School retention rate OR School days missed OR School attendance OR Intention to return) OR TI,AB(Antenatal OR Initiation of antenatal care OR Antenatal visit* OR Number of antenatal visits OR OR Postnatal visit* OR Post-delivery visit* OR Immuniz* OR Immunis* OR Vaccinat* OR Contracepti* OR Birth control OR Family planning OR Fertility control) OR TI,AB(Child development OR Positive parenting OR Parenting OR Parenting stress OR Parenting self-efficacy OR Discipline) OR TI,AB(Parenting behav* OR Responsive parenting OR Sensitiv* OR Sensitive parenting OR Positive interactions with child OR Discipline OR Behav* management) OR TI,AB(Victimi* OR Relationship conflict OR Relationship violence OR Dating violence OR Coercive sex OR Gender-based violence OR GBV OR Intimate partner violence OR Domestic violence OR Domestic abuse OR Relationship abuse OR Verbal abuse OR Psychological abuse OR Physical abuse) OR TI,AB(Adher* OR ARV initiation OR ARV discontinuation OR ARV OR ART OR Antiretroviral Treatment OR ARV adherence OR Viral load OR Viral suppression OR CD4 OR Medication possession ratio OR Pill count OR Tablet count OR Electronic monitoring of adherence) |
| 12 | Stress disorder OR Stress OR PTSD OR Post traumatic stress disorder OR Post-traumatic stress disorder OR Post-trauma* OR Post trauma* OR Psychological trauma OR Trauma* OR Complex PTSD |
| 13 | (1 AND (2 OR 3 OR 4 OR 5 OR 6 OR 7 OR 8) AND 9 AND 10 AND 11) OR 1 AND (2 OR 3 OR 4) AND 9 AND 10 AND 11 AND 12 |

**ERIC**

| 1 | AB(Adolescen* OR Juvenile* OR Student OR Pupil OR Learner OR Teen* OR Young adult OR Young people OR Youth OR Young wom* OR Young men OR Young man OR Pubescent) OR TI(Adolescen* OR Juvenile* OR Student OR Pupil OR Learner OR Teen* OR Young adult OR Young people OR Youth OR Young wom* OR Young men OR Young man OR Pubescent) | |
| --- | --- | --- |
| 2 | AB(Violen* OR Conflict) OR TI(Violen* OR Conflict) | |
| 3 | AB(Poverty OR Food insecur* OR Asset poor OR Asset index OR Disadvantaged OR Homeless OR Indigent OR Low income OR Low-income OR Poor OR Breadline OR Depriv* OR Extreme poverty OR Impoverished OR Destitute) OR TI(Poverty OR Food insecur* OR Asset poor OR Asset index OR Disadvantaged OR Homeless OR Indigent OR Low income OR Low-income OR Poor OR Breadline OR Depriv* OR Extreme poverty OR Impoverished OR Destitute) | |
| 4 | AB(Refugee OR Forced migrant OR Displace* OR Migrant OR Asylum seeker OR Persecution OR Civil disruption OR Conflict OR Humanitarian OR Political unrest OR Emergency OR Natural Disaster OR Fire OR Wildfire OR Hurricane OR Tornado OR Flood OR Tsunami OR Monsoon OR Earthquake OR Volcano OR Sandstorm OR Storm OR Landslide OR Mudslide OR Heatwave OR Cyclone OR Blizzard OR Tropical storm OR Drought OR Famine OR War) OR TI(Refugee OR Forced migrant OR Displace* OR Migrant OR Asylum seeker OR Persecution OR Civil disruption OR Conflict OR Humanitarian OR Political unrest OR Emergency OR Natural Disaster OR Fire OR Wildfire OR Hurricane OR Tornado OR Flood OR Tsunami OR Monsoon OR Earthquake OR Volcano OR Sandstorm OR Storm OR Landslide OR Mudslide OR Heatwave OR Cyclone OR Blizzard OR Tropical storm OR Drought OR Famine OR War) | |
| 5 | AB(Perinatal OR Prenatal OR Postnatal OR Antenatal OR Peri-natal OR Pre-natal OR Post-natal OR Ante-natal OR Mother OR Father OR Parent* OR Expecting OR Expectant OR Pregnan* OR Maternal OR Paternal) OR TI(Perinatal OR Prenatal OR Postnatal OR Antenatal OR Peri-natal OR Pre-natal OR Post-natal OR Ante-natal OR Mother OR Father OR Parent* OR Expecting OR Expectant OR Pregnan* OR Maternal OR Paternal) | |
| 6 | AB(HIV OR AIDS) OR TI(HIV OR AIDS) | |
| 7 | AB(Depress* OR Anxiety OR Anxious OR Internali* OR Sad* OR Mood OR Affect* OR Dysphoria OR Emotion*) OR TI(Depress* OR Anxiety OR Anxious OR Internali* OR Sad* OR Mood OR Affect* OR Dysphoria OR Emotion*) | |
| 8 | AB(Aggress* OR Violen* OR Conflict OR Conduct OR Externali* OR Disruptive OR oppositional OR Impulse control OR Peer problems OR Antisocial* OR Anti-social OR Fight* OR Bully* OR Anger* OR Rage) OR TI(Aggress* OR Violen* OR Conflict OR Conduct OR Externali* OR Disruptive OR oppositional OR Impulse control OR Peer problems OR Antisocial* OR Anti-social OR Fight* OR Bully* OR Anger* OR Rage) | |
| 9 | AB(Education OR Indicated OR Intervention OR Prevent* OR Program* OR Promot* OR Support OR Targeted OR Therap* OR Training OR Treatment OR Universal OR Ccbt OR Cognitive Behav* OR CBT OR (Social N2 emotional learning) OR Psychosocial OR Psychoeduc* OR Psycho-educ* OR Interpersonal OR Life Skill*) OR TI(Education OR Indicated OR Intervention OR Prevent* OR Program* OR Promot* OR Support OR Targeted OR Therap* OR Training OR Treatment OR Universal OR Ccbt OR Cognitive Behav* OR CBT OR (Social N2 emotional learning) OR Psychosocial OR Psychoeduc* OR Psycho-educ* OR Interpersonal OR Life Skill*) | |
| 10 | AB(Randomi* OR Randomly OR RCT OR Trial OR Arms OR Double blind OR double-blind OR Factorial OR "control group" OR "controlled trial") OR TI(Randomi* OR Randomly OR RCT OR Trial OR Arms OR Double blind OR double-blind OR Factorial OR "control group" OR "controlled trial") | |
| 11 | AB(Well-being OR Wellbeing OR Well being OR Life satisfaction OR Quality of life OR Positive psychology OR Self-concept OR self concept OR Self-esteem OR Self esteem OR Self control OR Self-control OR Self-efficacy OR Self efficacy) OR TI(Well-being OR Wellbeing OR Well being OR Life satisfaction OR Quality of life OR Positive psychology OR Self-concept OR self concept OR Self-esteem OR Self esteem OR Self control OR Self-control OR Self-efficacy OR Self efficacy) OR AB(Mental function* OR Individual function* OR Social Function* OR Emotion* Function* OR Resilienc* OR Coping OR Problem Solv* OR Relationship skills OR Interpersonal skills OR Communicat* OR Emotion* regulat* OR Mental health) OR TI(Mental function* OR Individual function* OR Social Function* OR Emotion* Function* OR Resilienc* OR Coping OR Problem Solv* OR Relationship skills OR Interpersonal skills OR Communicat* OR Emotion* regulat* OR Mental health) OR AB(Depress* OR Anxiety OR Anxious OR Internali* OR Sad* OR Mood OR Affect* OR Dysphoria OR Emotion*) OR TI(Depress* OR Anxiety OR Anxious OR Internali* OR Sad* OR Mood OR Affect* OR Dysphoria OR Emotion*) OR AB (Conduct OR Oppositional behav* OR Oppositional defiant OR ODD) OR TI (Conduct OR Oppositional behav* OR Oppositional defiant OR ODD) OR AB(Self-harm OR Self harm OR Cutting OR Suicid*) OR TI(Self-harm OR Self harm OR Cutting OR Suicid*) OR AB(Alcohol OR Intoxication OR drink* OR Drunk* OR Marijuana OR marihuana OR hashish OR THC OR Cannabis OR Opioid* OR Opiate* OR morphine OR heroin OR methadone OR diamorphine OR diacetylmorphine OR fentanyl OR stimulant* OR ecstasy OR MDMA OR methylenedioxymethamphetamine OR methamphetamine OR amphetamine OR cocaine OR Substance abuse OR Substance use OR Substance dependence OR Substance addiction OR Tobacco OR Cigarette* OR Smoking or Drug*) OR TI(Alcohol OR Intoxication OR drink* OR Drunk* OR Marijuana OR marihuana OR hashish OR THC OR Cannabis OR Opioid* OR Opiate* OR morphine OR heroin OR methadone OR diamorphine OR diacetylmorphine OR fentanyl OR stimulant* OR ecstasy OR MDMA OR methylenedioxymethamphetamine OR methamphetamine OR amphetamine OR cocaine OR Substance abuse OR Substance use OR Substance dependence OR Substance addiction OR Tobacco OR Cigarette* OR Smoking OR Drug*) OR AB(Aggress* OR Violen* OR Conflict OR Conduct OR Externali* OR Disruptive OR oppositional OR Impulse control OR Peer problems OR Antisocial* OR Anti-social OR Fight* OR Bully* OR Anger* OR Rage OR Arrest* OR Incarcerat* OR Criminal behav*) OR TI(Aggress* OR Violen* OR Conflict OR Conduct OR Externali* OR Disruptive OR oppositional OR Impulse control OR Peer problems OR Antisocial* OR Anti-social OR Fight* OR Bully* OR Anger* OR Rage OR Arrest* OR Incarcerat* OR Criminal behav*) OR AB(Condom* OR Safe sex OR Unsafe sex OR Protected sex OR Responsible sex OR Unprotected sex OR Safe intercourse OR Unsafe intercourse OR Protected intercourse OR Unprotected intercourse OR Multiple sexual partners OR Multiple partners OR Number of partners OR Early sexual debut OR Early sexual initiation OR Age at first sex* OR Early intercourse OR Early first sex OR Concurrent partners) OR TI(Condom* OR Safe sex OR Unsafe sex OR Protected sex OR Responsible sex OR Unprotected sex OR Safe intercourse OR Unsafe intercourse OR Protected intercourse OR Unprotected intercourse OR Multiple sexual partners OR Multiple partners OR Number of partners OR Early sexual debut OR Early sexual initiation OR Age at first sex* OR Early intercourse OR Early first sex OR Concurrent partners) OR AB(School OR School enrol* OR School drop* OR School retention rate OR School days missed OR School attendance OR Intention to return OR) OR TI(School OR School enrol* OR School drop* OR School retention rate OR School days missed OR School attendance OR Intention to return) OR AB(Antenatal OR Initiation of antenatal care OR Antenatal visit* OR Number of antenatal visits OR Postnatal visit* OR Post-delivery visit* OR Immuniz* OR Immunis* OR Vaccinat* OR Contracepti* OR Birth control OR Family planning OR Fertility control) OR TI(Antenatal OR Initiation of antenatal care OR Antenatal visit* OR Number of antenatal visits OR Postnatal visit* OR Post-delivery visit* OR Immuniz* OR Immunis* OR Vaccinat* OR Contracepti* OR Birth control OR Family planning OR Fertility control) OR AB(Child development OR Positive parenting OR Parenting OR Parenting stress OR Parenting self-efficacy OR Discipline) OR TI(Child development OR Positive parenting OR Parenting OR Parenting stress OR Parenting self-efficacy OR Discipline) OR AB(Parenting behav* OR Responsive parenting OR Sensitiv* OR Sensitive parenting OR Positive interactions with child OR Discipline OR Behav* management) OR TI(Parenting behav* OR Responsive parenting OR Sensitiv* OR Sensitive parenting OR Positive interactions with child OR Discipline OR Behav* management) OR AB(Victimi* OR Relationship conflict OR Relationship violence OR Dating violence OR Coercive sex OR Gender-based violence OR GBV OR Intimate partner violence OR Domestic violence OR Domestic abuse OR Relationship abuse OR Verbal abuse OR Psychological abuse OR Physical abuse) OR TI(Victimi* OR Relationship conflict OR Relationship violence OR Dating violence OR Coercive sex OR Gender-based violence OR GBV OR Intimate partner violence OR Domestic violence OR Domestic abuse OR Relationship abuse OR Verbal abuse OR Psychological abuse OR Physical abuse) OR AB(Adher* OR ARV initiation OR ARV discontinuation OR ARV OR ART OR Antiretroviral Treatment OR ARV adherence OR Viral load OR Viral suppression OR CD4 OR Medication possession ratio OR Pill count OR Tablet count OR Electronic monitoring of adherence) OR TI(Adher* OR ARV initiation OR ARV discontinuation OR ARV OR ART OR Antiretroviral Treatment OR ARV adherence OR Viral load OR Viral suppression OR CD4 OR Medication possession ratio OR Pill count OR Tablet count OR Electronic monitoring of adherence) | |
| 12 | AB(Stress disorder OR Stress OR PTSD OR Post traumatic stress disorder OR Post-traumatic stress disorder OR Post-trauma* OR Post trauma* OR Psychological trauma OR Trauma* OR Complex PTSD) OR TI(Stress disorder OR Stress OR PTSD OR Post traumatic stress disorder OR Post-traumatic stress disorder OR Post-trauma* OR Post trauma* OR Psychological trauma OR Trauma* OR Complex PTSD) | |
| 13 | (S1 AND (S2 OR S3 OR S4 OR S5 OR S6 OR S7 OR S8) AND S9 AND S10 AND S11) OR (S1 AND (S2 OR S3 OR S4) AND S9 AND S10 AND S11 AND S12) |  |
