# Supplementary figures and images for: Psychosocial interventions targeting mental health in pregnant adolescents and adolescent parents: a systematic review
Source: Reprod Health. 2020 May 14;17:65. doi: 10.1186/s12978-020-00913-y (PMC7227359; doi:10.1186/s12978-020-00913-y)

**Supplemental file 2: Risk of bias assessments for all included studies**

**
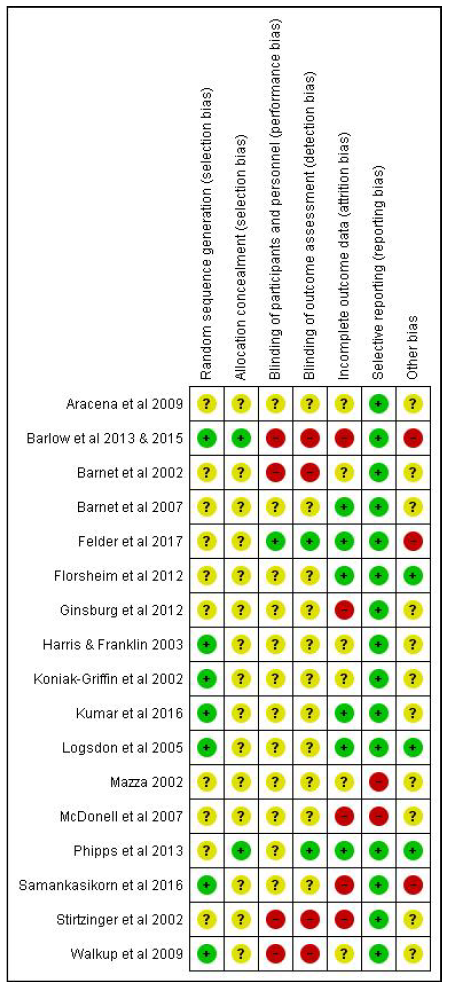
**

Supplement: Supplementary file 2 — Additional file 2. [file 12978_2020_913_MOESM2_ESM.docx]

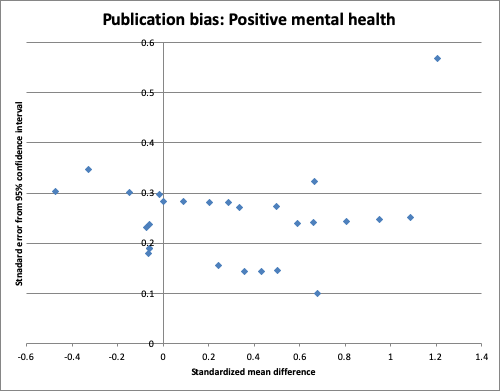


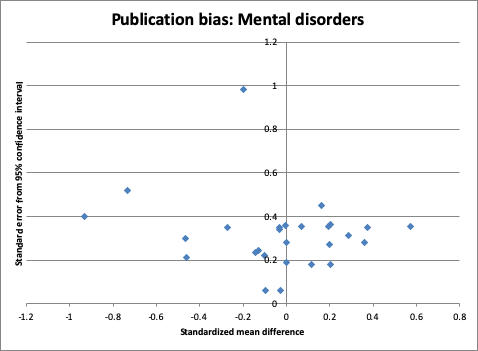

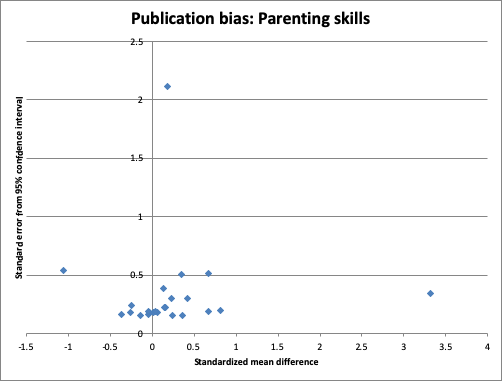


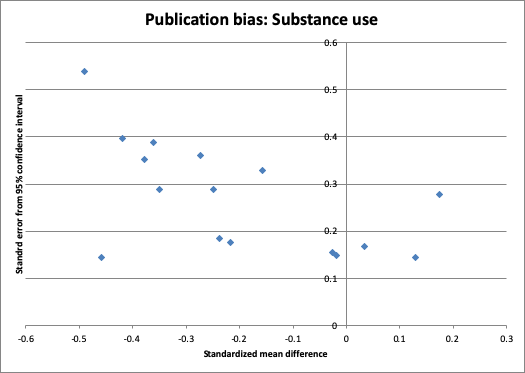

Supplement: Supplementary file 4 — Additional file 4. [file 12978_2020_913_MOESM4_ESM.docx]
