# Supplementary material for: Psychosocial interventions targeting mental health in pregnant adolescents and adolescent parents: a systematic review
Source: Reprod Health. 2020 May 14;17:65. doi: 10.1186/s12978-020-00913-y (PMC7227359; doi:10.1186/s12978-020-00913-y)
Supplement: Supplementary file 3 — Additional file 3. [file 12978_2020_913_MOESM3_ESM.docx]

**Forest plots: pregnant adolescent and adolescent parents**

**Effect sizes: Substance use outcomes**

**
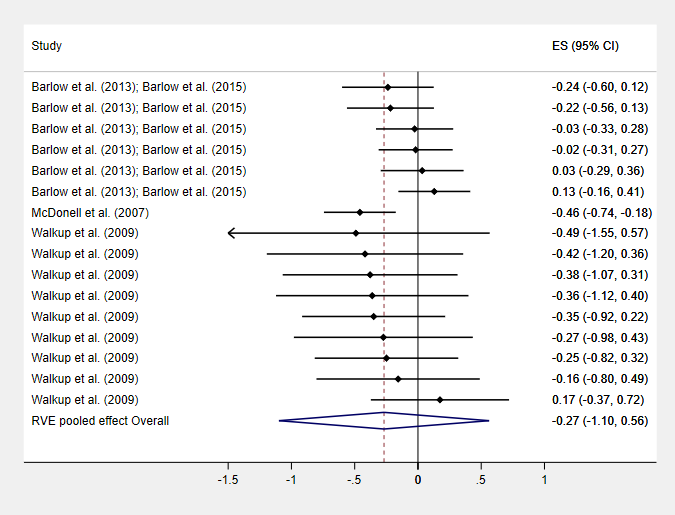
**

**Effect sizes: School attendance outcomes**

**
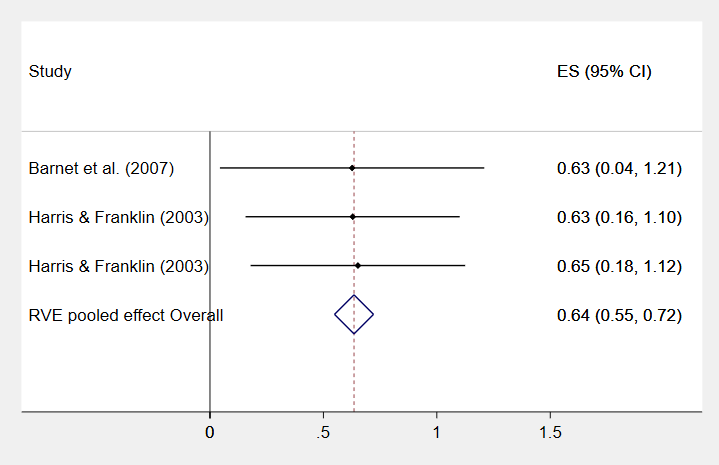
**

**Effect sizes: risky sexual and reproductive health behaviour outcomes**

**
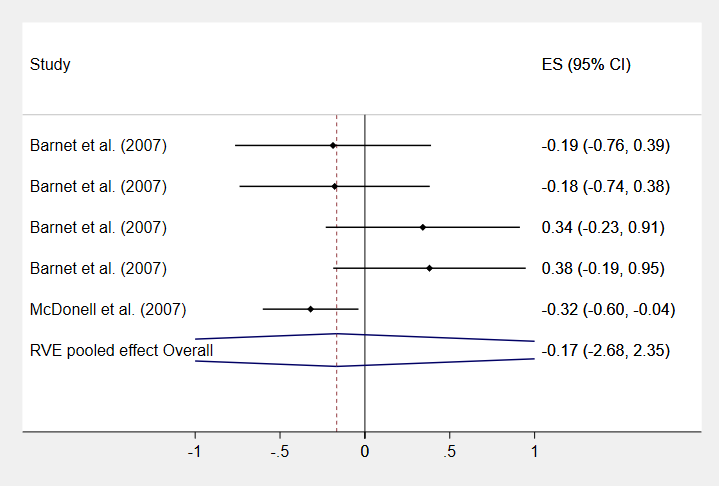
**

**Effect sizes: Positive mental health outcomes**

**
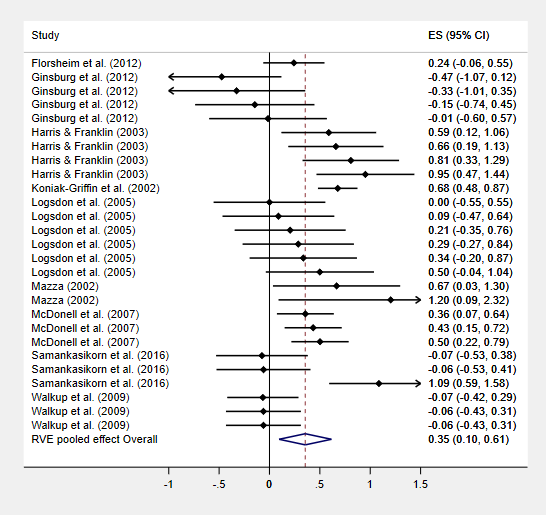
**

**Effect sizes: Parenting skills outcomes**

**
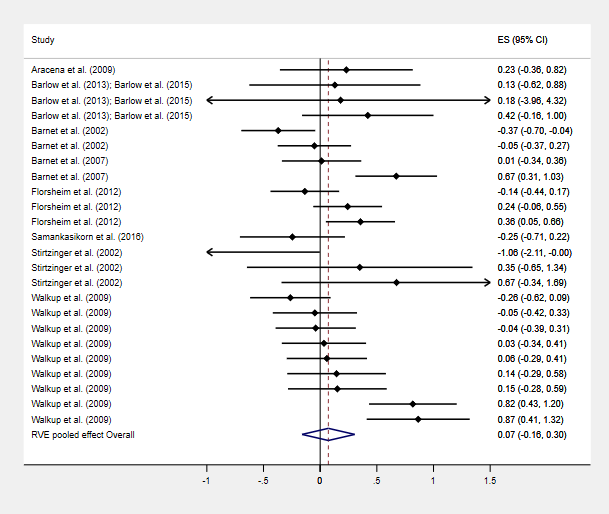
**

**Effect sizes: Mental disorders outcomes**

**
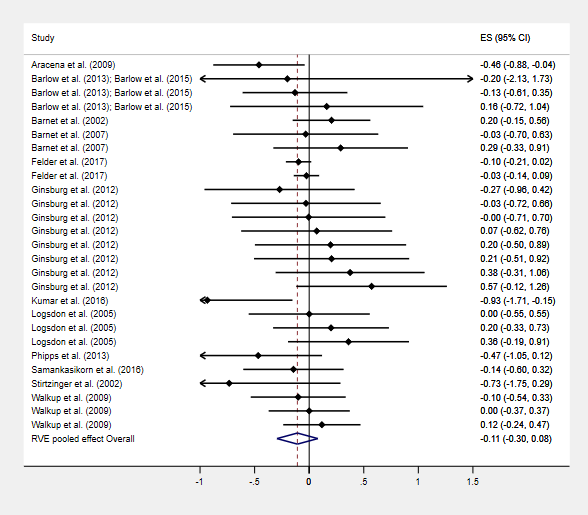
**

**Effect sizes: Adherence to antenatal and postnatal care outcomes**

**
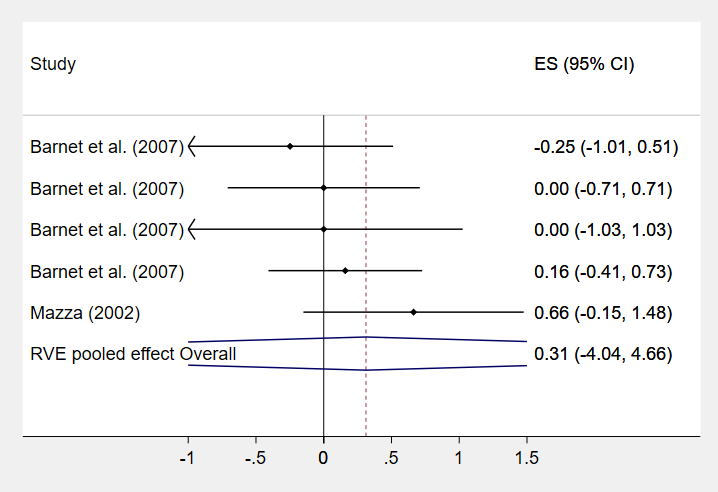
**
